# Mutations in starch *BRANCHING ENZYME 2a* suppress the traits caused by the loss of ISOAMYLASE1 in barley

**DOI:** 10.1007/s00122-024-04725-7

**Published:** 2024-08-31

**Authors:** Ryo Matsushima, Hiroshi Hisano, June-Sik Kim, Rose McNelly, Naoko F. Oitome, David Seung, Naoko Fujita, Kazuhiro Sato

**Affiliations:** 1 Institute of Plant Science and Resources, Okayama University, 2-20-1 Chuo, Kurashiki, Okayama 710-0046 Japan; 2 RIKEN Center for Sustainable Resource Science, Yokohama, 230-0045 Japan; 3 John Innes Centre, Norwich Research Park, Norwich,, NR4 7UH UK; 4 Department of Biological Production, Akita Prefectural University, Akita, 010-0195 Japan

## Abstract

**Key message:**

The *hvbe2a* mutations restore the starch-deficient phenotype caused by the *hvisa1* and *hvflo6* mutations in barley endosperm.

**Abstract:**

The genetic interactions among starch biosynthesis genes can be exploited to alter starch properties, but they remain poorly understood due to the various combinations of mutations to be tested. Here, we isolated two novel barley mutants defective in starch *BRANCHING ENZYME 2a* (*hvbe2a-1* and *hvbe2a-2*) based on the starch granule (SG) morphology. Both *hvbe2a* mutants showed elongated SGs in the endosperm and increased resistant starch content. *hvbe2a-1* had a base change in *HvBE2a* gene, substituting the amino acid essential for its enzyme activity, while *hvbe2a-2* is completely missing *HvBE2a* due to a chromosomal deletion. Further genetic crosses with barley *isoamylase1* mutants (*hvisa1)* revealed that both *hvbe2a* mutations could suppress defects in endosperm caused by *hvisa1*, such as reduction in starch, increase in phytoglycogen, and changes in the glucan chain length distribution. Remarkably, *hvbe2a* mutations also transformed the endosperm SG morphology from the compound SG caused by *hvisa1* to bimodal simple SGs, resembling that of wild-type barley. The suppressive impact was in competition with *floury endosperm 6* mutation (*hvflo6*), which could enhance the phenotype of *hvisa1* in the endosperm. In contrast, the compound SG formation induced by the *hvflo6 hvisa1* mutation in pollen was not suppressed by *hvbe2a* mutations. Our findings provide new insights into genetic interactions in the starch biosynthetic pathway, demonstrating how specific genetic alterations can influence starch properties and SG morphology, with potential applications in cereal breeding for desired starch properties.

**Supplementary Information:**

The online version contains supplementary material available at 10.1007/s00122-024-04725-7.

## Introduction

Starch, a glucose-based polymer from plants, is extensively utilized in various food and industrial sectors. Its water-insolubility and lack of osmotic activity make it an ideal substance for long-term storage in seeds, grains, and roots. Within these storage organs, starch forms semi-crystalline starch granules (SGs) produced in specialized plastids called amyloplasts (Gunning and Steer 1996). SGs vary in size and shape across different plant species and are broadly categorized as either compound or simple SGs (Tateoka 1962; Matsushima et al. 2013; Chen et al. 2021). Compound SGs consist of assemblies of smaller starch particles, whereas simple SGs are composed of single starch particles. In the case of rice (*Oryza sativa*) endosperm, the compound SGs measure about 10–20 μm in diameter and are made up of polyhedral starch particles, each measuring 3–8 μm (Matsushima et al. 2010). Meanwhile, the endosperm of barley (*Hordeum vulgare*) and wheat (*Triticum aestivum*) features two types of simple SGs, the smaller B-type (~ 5 μm diameter) and the larger A-type (10–30 μm diameter), both existing together within a single cell (Jane et al. 1994; Matsushima and Hisano 2019; Thieme et al. 2023). This type is referred to as bimodal simple SGs. The A-type granules begin to form in the early stages of grain development within amyloplasts, followed by the development of B-type granules in the same amyloplasts, which already contain A-type granules (Langeveld et al. 2000; Matsushima and Hisano 2019; Kamble et al. 2023).

The size and shape of SGs are influenced by specific enzymes involved in synthesizing amylopectin, the primary polymer component of SGs, which is made up of α-1,4-linked and α-1,6-branched glucose chains (Smith and Zeeman 2020; Tetlow and Bertoft 2020). Amylopectin synthesis involves three key reactions: the formation of α-1,4-glycosidic bonds by starch synthases (SSs), which elongate the glucose chains; the creation of α-1,6-glycosidic bonds by branching enzymes (BEs), introducing branches in the structure; and the removal of misplaced glucose branches by hydrolyzing α-1,6 linkages with starch debranching enzymes (DBEs) (Nakamura 2002; Pfister and Zeeman 2016). In the absence of appropriate trimming by DBEs, improperly positioned branches can prevent the formation of the semi-crystalline structure of amylopectin. Deficiency of one of the DBEs, ISOAMYLASE1 (ISA1), leads to the accumulation of phytoglycogen, a water-soluble α-glucan (Pan and Nelson 1984; Nakamura et al. 1997; Burton et al. 2002). Phytoglycogen is characterized by its extensive branching with short glucan chains and has a molecular weight significantly lower than that of amylopectin. In barley *ISA1* mutants (*hvisa1*), the endosperm forms compound SGs, contrasting with the typical bimodal simple SGs found in the wild type (Burton et al. 2002; Matsushima et al. 2023). Cereal mutants exhibiting defects in starch biosynthetic enzymes hold considerable interest in the food industry due to their capability to alter the physicochemical and nutritional properties of starch within the grain (Nakamura 2018). For example, mutations in the *ISA1* gene of maize lead to increased soluble sugars, instead of starch in the grains and have been utilized in breeding sweet corn varieties (Gonzales et al. 1976; Pan and Nelson 1984; James et al. 1995). Furthermore, mutants in the *BE* genes of rice and maize are noted to enhance the production of resistant starch, known for its resistance to digestion by amylase (Xia et al. 2011; Tsuiki et al. 2016; Chen et al. 2022). The intake of resistant starch changes the composition of gut microbiota in mice, marked by an increase in specific beneficial bacteria (Li et al. 2024). This contributes to the improvement in obesity by reducing lipid absorption, decreasing inflammation, and enhancing intestinal barrier strength (Blaak et al. 2020).

Recent studies have identified several non-enzymatic proteins that directly interact with starch biosynthetic enzymes and also affect SG shape and size. These proteins include the *Arabidopsis thaliana* PROTEIN TARGETING TO STARCH (PTST) 1 to 3, the rice PTST2 ortholog, FLOURY ENDOSPERM 6 (FLO6) and its barley and wheat orthologs, HvFLO6 and B-GRANULE CONTENT1 (BGC1), respectively (Peng et al. 2014; Seung et al. 2015, 2017; Saito et al. 2018; Chia et al. 2020). These proteins possess Carbohydrate-Binding Module 48 (CBM48) domains, known for their affinity towards binding starch and maltooligosaccharides (Peng et al. 2014; Seung et al. 2015, 2017). CBM48s are also found in the sequences of BEs and DBEs, but not in SSs (Pfister and Zeeman 2016). CBM48s probably facilitate the efficient transfer of the substrate to starch biosynthetic enzymes. In the barley *hvflo6* mutant (also known as *Franubet*), the endosperm develops an unusual mix of simple and compound SGs (DeHaas BW and Goering KJ 1983; Chung T-Y 2001; Suh et al. 2004; Matsushima et al. 2023). In wheat, the reduced expression of BGC1 decreases B-type granule content in endosperm (Chia et al. 2017, 2020). In addition, the non-enzymatic protein, LIKE EARLY STARVATION (LESV), was recently reported to be involved in starch biosynthesis in Arabidopsis and rice (Liu et al. 2023; Dong et al. 2024; Yan et al. 2024). In rice, LESV can interact with FLO6 and mediate the localization of starch biosynthetic enzymes to starch (Dong et al. 2024; Yan et al. 2024).

Studies on the genetic interactions among mutations in starch biosynthesis genes have mainly focused on rice and maize (Ferguson et al. 1979; Toyosawa et al. 2016; Lee et al. 2017; Ida et al. 2021; Nagamatsu et al. 2022). In barley, the reduction in starch content and the corresponding increase in phytoglycogen in the *hvisa1* grains is further enhanced by the *hvflo6* mutation (Matsushima et al. 2023). The enhancement exceeds just an additive effect, indicating potential genetic interactions between the two mutations. The genetic interaction between *hvflo6* and *hvisa1* is also observed in the pollen grain, which accumulates starch like the endosperm of cereals. In both wild-type and *hvisa1* pollen, simple, rod-shaped SGs predominantly are developed. In the *hvflo6* pollen, the number of compound SGs is increased, and this increase is significantly pronounced in the *hvflo6 hvisa1* double mutant (Matsushima et al. 2023).

In our previous study, we reported on the *hvisa1* and *hvflo6* mutants in barley, particularly in the elite Japanese malting barley cultivar ‘Haruna Nijo’, using the rapid, simple observation method of SGs (Matsushima et al. 2023). Unlike other procedures, this method does not require chemical fixation and resin embedding of samples, making it suitable for dealing with large number of samples (Matsushima et al. 2010). In this study, we report newly identified barley mutants, *hvbe2a-1* and *hvbe2a-2*, which develop elongated SGs in endosperm, that are not present in the wild type. Both mutants had genetic lesions in the *HvBE2a* gene coding a major BE in barley endosperm. Our analysis using triple and double mutants of *hvisa1*, *hvflo6* and *hvbe2a* revealed that *hvbe2a* mutation has a suppressive effect against *hvisa1* mutation in endosperm. *hvisa1* mutation altered the SG morphology from a bimodal to a compound type, and the addition of *hvbe2a* mutation reversed the SG morphology back to the bimodal form. In contrast to the endosperm, the suppressive effect of *hvbe2a* was not observed in pollen. These findings contribute to our understanding of the genetic network in starch biosynthesis and provide new insights into the determinants of SG morphology.

## Materials and methods

### Plant material and growth conditions

Barley cultivar’Haruna Nijo’ was provided from NBRP-Barley (http://earth.nig.ac.jp/~dclust/cgi-bin/index.cgi?lang=en). *hvisa1-3* and *hvflo6-2* were previously reported (Matsushima et al. 2023). *hvbe2a-1* and *hvbe2a-2* were isolated from the same screening described previously (Matsushima et al. 2023). The mutants were crossed with cv Haruna Nijo, and the progeny exhibiting *hvbe2a* phenotype were used in this study. Double and triple mutants were generated by artificially crossing individual mutants, and multiple homozygous individuals were selected from the F2 progeny using PCR-based genotyping. Barley plants were grown at 22 °C/18 °C in a growth cabinet (NK Systems, LPH-411S) or around 23 °C continuously at 16 h day/8 h night conditions. The shoot weight and number of tillers were measured 28 days after germination.

### Observation of SGs by thin-section microscopy with Technovit 7100 Resin

The preparation of Technovit sections to observe SGs in the endosperm is described previously (Matsushima et al. 2014).

### Purification of starch and quantification of starch granule size distributions

Starch purification was essentially conducted following Kamble et al. (2023). A grain was ground using the Multi-beads Shocker MB2000 (YASUI KIKAI, Japan), and the sample was resuspended in 2 mL water. The homogenates were filtered through a 100-μm nylon mesh, then centrifuged at 3,000 × *g* for 5 min. The pellet was resuspended in 2 mL water. The starch suspension on a 5-mL cushion of 90% (v/v) Percoll in 50 mM Tris–HCl (pH 8) was centrifuged at 2,500 × *g* for 15 min. The pellet was washed twice in 50 mM Tris–HCl (pH 6.8), 10 mM EDTA, 4% SDS (v/v), 10 mM DTT and then three times in water. Finally, the purified starch was washed with ethanol twice and dried.

To quantify starch granule size distributions, the purified starch was suspended in Isoton II electrolyte solution (Beckman Coulter, Indianapolis), and particle sizes were measured using a Multisizer 4e Coulter Counter (Beckman Coulter) fitted with a 70-μm aperture tube. A minimum of 100,000 particles was measured per sample. These data were used to produce relative percent volume vs. diameter plots. A mixed bimodal distribution (normal and lognormal distributions) was fitted to the relative percent volume vs. diameter plots (Python script available at: https://github.com/DavidSeungLab/Coulter-Counter-Data-Analysis) to calculate mean diameters of A- and B-type granules by fitting normal curves for A-type and lognormal curves for B-type to the granule size distribution traces (Hawkins et al. 2021).

### Preparation of antibodies against HvBE2a, HvBE2b and HvBE1

All of the antibodies in this study were raised against synthetic peptides. Anti-HvBE2a antibodies were raised against AAAPGKVLVPDGESDDL (amino acid position from 52 to 68 of HvBE2a) and VDYFTTEHPHDNRPRS (amino acid position from 789 to 804 of HvBE2a). The two antibodies were named anti-HvBE2a-N and anti-HvBE2a-C, respectively. Anti-HvBE2b antibody was raised against AGGPSGEVMI (amino acid position from 58 to 67 of HvBE2b). Anti-HvBE1 antibody was raised against KRGINFVFRSPDKDNK (amino acid position from 810 to 825 of HvBE1). The immunization of rabbits and purification of antibodies were outsourced to Cosmo Bio (Tokyo, Japan) or Eurofins Genomics (Tokyo, Japan).

### Immunoblot analysis following SDS-PAGE

Developing grains at 14 days after awn emergence (DAA) were cultivated and stored at − 80 °C before use. The grain was mixed with 150 μL of ice-cold grinding solution (50 mM imidazole–HCl [pH 7.4], 8 mM MgCl_2_, 12.5% [v/v] glycerol), and then crushed using plastic pestles. The homogenates were centrifugated at 16,000 × *g* at 4 °C for 5 min. The supernatant (50 μL) was mixed with 50 μL of SDS-sample buffer (2% [w/v] SDS, 100 mM Tris–HCl [pH 6.8], 2% [v/v] 2-mercaptoethanol, 40% [v/v] glycerol) and incubated at 98 °C for 10 min. 8 μL was subjected to SDS-PAGE, and proteins were transferred electrophoretically to a polyvinylidene difluoride membrane (Millipore). The membrane was then incubated in Phosphate-buffered saline (pH 7.4) plus 0.05% (v/v) Tween–20 with the antibodies for 1 h. Dilutions of the antibodies were 1:3,000—5,000 (v/v). The secondary antibody was an Anti-Rabbit IgG, HRP-Linked Whole Ab Donkey (Cytiva, NA934V), which was diluted (1:5,000). The immunoreactive bands were detected with Immobilon Crescendo Western HRP substrate (Millipore, WBLUR0500).

### Quantification of starch and phytoglycogen

Total starch and phytoglycogen quantification in a grain are described previously (Matsushima et al. 2023). The Resistant Starch Assay Kit (Megazyme, K-RSTAR) was used according to the manufacturer’s instructions to measure the amount of resistant starch as a percentage of total starch.

### Glucan chain length distribution of total α-glucan in grain

The procedures to detect the glucan-chain-length distribution of total α-glucan are the same as in our previous study (Matsushima et al. 2023).

### Genotyping of mutations

The base change of *hvbe2a-1* mutation was detected by the derived cleaved-amplified polymorphic sequence primers: 5’-TTAGGTGGCGAAGGCTATCTTAATTCCATG-3’ and 5’-GTTCAAATTACAATAAATCGCAACC-3’. The PCR conditions were as follows: 94 °C for 2 min and 35 cycles of 94 °C for 30 s, 53 °C for 45 s, and 68 °C for 1 min. The PCR product was digested with *Nco*I, and PCR products were subsequently separated by 15% polyacrylamide gel electrophoresis (PAGE) and detected with ethidium bromide staining. In the case of wild type, a PCR product (109 bp) was digested into 83 and 26 bp. In the case of *hvbe2a-1*, the PCR product was not digested. *hvisa1-3* and *hvflo6-2* mutation sites were detected according to the previous paper (Matsushima et al. 2023).

### Genome sequencing of *hvbe2a-2*

High-molecular-weight DNA was isolated from leaf material of *hvbe2a-2* seedlings using NucleoBond HMW DNA (Takara). A total of 0.5 μg of the isolated DNA underwent sequencing library preparation using the MGIEasy FS PCR-Free DNA library Prep Kit (MGI Tech, Shenzhen, China). Whole-genome sequencing was performed on the DNBSEQ-G400RS platform (MGI Tech) by a commercial vendor (Genome-Lead Co., Kagawa, Japan), yielding 375.9 million reads (2 × 150 nucleotides) of the *hvbe2a-2* genome. The genomic sequence reads of barley cv Haruna Nijo (1.5 billion reads, 2 × 100 nucleotides) were obtained from a previous report (Sato et al. 2016). These sequence reads underwent quality control and were trimmed using Trimmomatic version 0.39 (Bolger et al. 2014), then mapped to the reference sequence assembly of Haruna Nijo (Sakkour et al. 2022) using bwa-mem version 0.7.17-r1188 (Li and Durbin 2009) with the default parameters. Only uniquely mapped reads were retained, then the read depth for genomic regions were assessed and visualized using IGV version 2.16.2 (Robinson et al. 2011).

### Detection of branching enzyme activity in the developing endosperm following Native-PAGE﻿

After thawing the harvested grains at 14 DAA, the removal of the embryo portion was followed by applying gentle pressure to the opposite side, facilitating the extraction of the endosperm from the grain. The endosperm was homogenized in the ice-cold grinding solution (80 μL), and then crushed using plastic pestles. The homogenates were subjected to centrifugation at 16,000 × *g* at 4 °C for 5 min. Protein concentration of the supernatant was determined using a Bradford Protein Assay Kit (Takara, Japan, T9310A). The supernatant was mixed with sample buffer (400 mM Tris–HCl [pH 7.0], 33% [v/v] glycerol) to adjust the protein concentration to 1 μg/μL. Proteins (7.5 μg) were subjected to Native-PAGE. BE activity staining was assessed using a gel containing 0.0001% oyster glycogen (Yamanouchi and Nakamura 1992). The immunoblot analysis following the Native-PAGE is identical to the analysis following SDS-PAGE.

### Observation of SGs in pollen grains﻿

To stain SGs in mature pollen, anthers at 3–5 DAA were disrupted with forceps in 120-times diluted Lugol solution on a glass cover slide. The released pollen was squashed by putting gentle pressure on a coverslip to release SGs from pollen vegetative cells. The released SGs were observed with a microscope (Olympus, BX53). For the quantification of compound SGs in pollen, the SGs within the 30 μm × 30 μm field of view were classified into simple and compound forms through visual inspection.

### Isolation of pollen grains and protein extraction from pollen﻿

Anthers, at 3 DAA, were collected in a 1.5 mL plastic tube and stored at − 80 °C until use. The anthers were mixed with the ice-cold grinding solution (1 mL) and chopped using a surgical scissor (Hammacher, HSB022-12). The homogenates were then filtered through a 100-μm nylon mesh to remove the debris other than pollen. The flow through was centrifuged at 1,000 × *g* at 4 °C for 1 min. The resulting pellet of pollen grains was resuspended in the ice-cold grinding solution (500 μL) and centrifuged again. The pollen pellet was resuspended once more in the ice-cold grinding solution (50 μL) and disrupted using plastic pestles in the tube. The homogenates were centrifuged at 10,000 × *g* at 4 °C for 2 min. The supernatant was recovered in another tube and protein concentration was measured using the Bradford Protein Assay kit. The supernatant was mixed with grinding solution and SDS-sample buffer to adjust the protein concentration to 0.5 μg/μL and heated immediately at 98 °C for 10 min. The resulting protein extract containing 5 μg of total protein was subjected to SDS-PAGE and immunoblot analysis.

## Results

### Barley mutants with elongated starch granules in endosperm

**Fig. 1:**
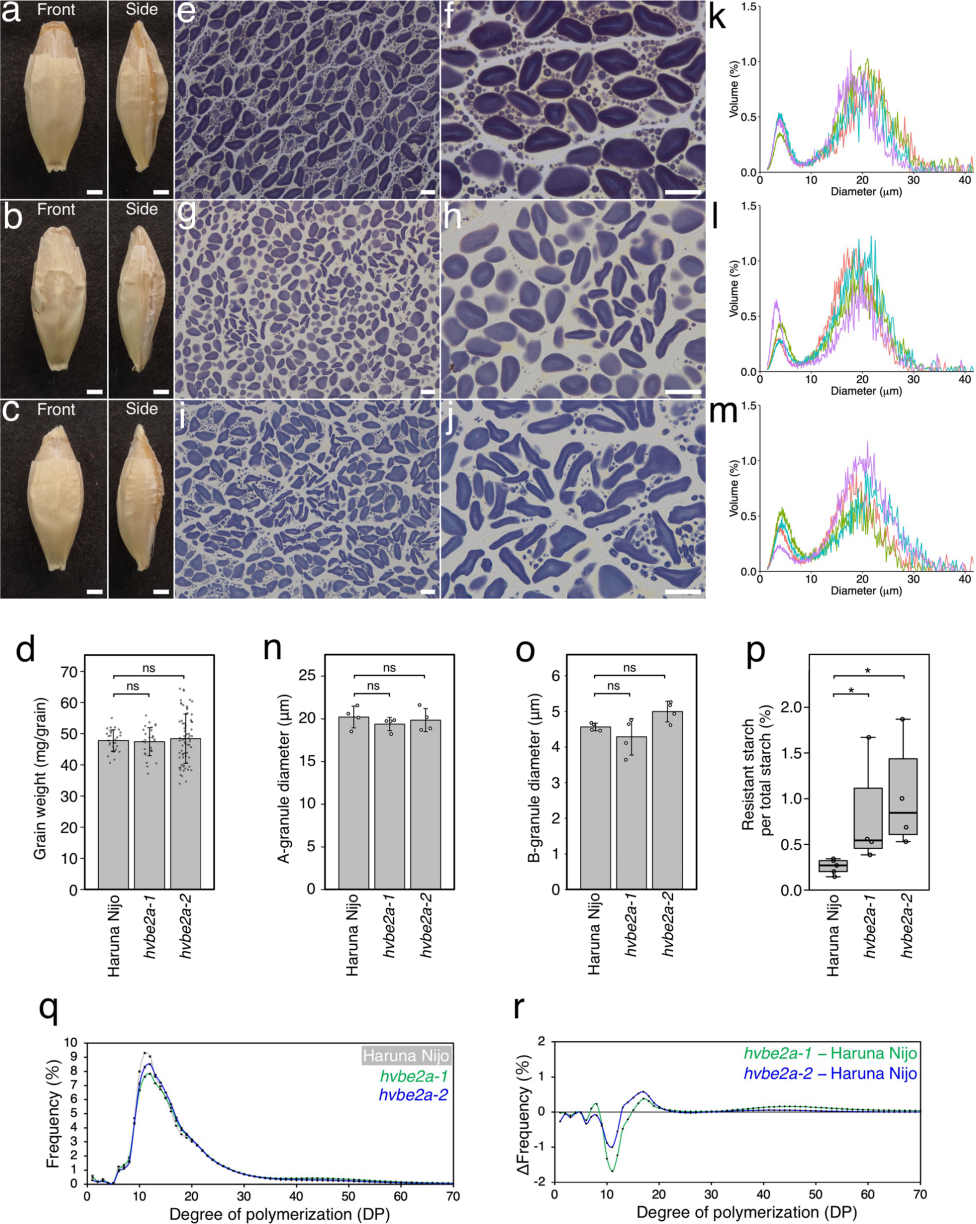
Isolation of barley mutants with elongated starch granules. **a**–**c** Mature grains of Haruna Nijo **a** and *hvbe2a-1* **b** and *hvbe2a-2* **c**. Front and side views are shown. Bars = 1 mm. **d** Single grain weight of Haruna Nijo, *hvbe2a-1* and *hvbe2a-2* at the mature stage (n = 30, 30, 70, respectively). Statistical comparisons were performed using Welch’s *t*-test (ns, not significant at *p* = 0.05). **e**–**j** Iodine-stained thin sections of endosperm cells of Haruna Nijo (e and f), *hvbe2a-1* (g and h), and *hvbe2a-2* (i and j). Bars = 20 μm. **k**–**m** Granule size distributions of Haruna Nijo, *hvbe2a-1* and *hvbe2a-2*, respectively. The relative percent volume of each diameter was determined using a Coulter Counter (n = 4 each). The graphs are displayed by overlaying the data from four biological replicates with different colors. **n**–**o** The average diameter of A- and B-type granules, respectively, extracted from the relative percent volume vs. diameter plots (k-m) by fitting a bimodal mixed normal and lognormal distribution. **p** Resistant starch amount in total starch of *hvbe2a* grains (n = 4 each). Statistical comparisons were performed using Wilcoxon rank sum test (*, *p* < 0.05). **q** Glucan chain-length distribution of α-glucans in *hvbe2a* mutant grains. *hvbe2a-1*, *hvbe2a-2* and Haruna Nijo are indicated by green, blue and grey lines, respectively. Data are given as means ± SD. All data were obtained from at least three independent grains. **r** Difference plot corresponding to the glucan-chain-length distribution profile presented in (q). The value for each chain length of Haruna Nijo was subtracted from that of the *hvbe2a* mutants

In our screen for barley mutants with altered SG morphology, we examined endosperm SGs in a barley sodium-azide-mutagenized population derived from the Japanese elite malting barley cultivar Haruna Nijo (Matsushima et al. 2023). We isolated mutants, namely *hvbe2a-1* and *hvbe2a-2*, which exhibited elongated SGs in the endosperm (Fig. 1). Subsequent sequencing analysis identified the causative mutations are in the *HvBE2a* gene of both mutants (described in a subsequent section); for clarity, we will use the *hvbe2a-1* and *hvbe2a-2* nomenclature to refer to mutants of interest. Haruna Nijo refers to the wild-type reference in this paper.

The mature grains of these mutants displayed a similar appearance to those of the wild type (Fig. 1a–c). The grain weight of the *hvbe2a-1* and *hvbe2a-2* were almost the same as those of Haruna Nijo (Fig. 1d). The dimensions of grains, including length, width, and thickness, from the *hvbe2a-1* mutant were almost the same as those of Haruna Nijo. Certain measurements from the *hvbe2a-2* mutant showed small changes compared to Haruna Nijo, but they were not consistently significant for both *hvbe2a-1* and *hvbe2a-2* (Supplementary Fig. 1a–c). This means that they are not caused by the mutation per se.

Iodine-stained Technovit thin sections of the endosperm revealed that Haruna Nijo developed typical bimodal SGs (Fig. 1e, f). In contrast, in *hvbe2a-1* (Fig. 1g, h) and *hvbe2a-2* (Fig. 1i, j) mutants, some SGs displayed elongated shapes. Coulter Counter analysis demonstrated that SGs from both Haruna Nijo and the *hvbe2a* mutants exhibited a similar bimodal distribution (Fig. 1k–m). The average diameters of A- and B-type SGs were not significantly different between Haruna Nijo and the *hvbe2a* mutants (Fig. 1n, o). The minimal changes on the distribution curves detected by the Coulter Counter analysis indicates that the elongated SGs in the *hvbe2a* mutants were either similar in volume to normal A-type granules, or not the majority. Next, we measured the amount of resistant starch in the *hvbe2a* mutant grains. Both *hvbe2a-1* and *hvbe2a-2* grains contained higher amount of resistant starch compared to Haruna Nijo; however, significant variation was observed among the biological replicates (Fig. 1p). The glucan chain length distribution of α-glucan from *hvbe2a* grains showed subtle differences compared to Haruna Nijo (Fig. 1q). The differential plot showed that both *hvbe2a* mutants had decreased glucose chains at degree of polymerization (DP) 11, while showing an increase in the frequency of chains at DP 17, relative to Haruna Nijo (Fig. 1r).

Plant appearance at 28 days after germination showed no significant difference in tiller numbers and shoot weights among Haruna Nijo, *hvbe2a-1* and *hvbe2a-2* mutants (Supplementary Fig. 2a–e). The panicle appearance of *hvbe2a* mutants was similar to that of Haruna Nijo at 20 DAA and mature stage (Supplementary Fig. 2f–k).

### Genetic lesion in *BRANCHING ENZYME 2a* gene in barley

**Fig. 2:**
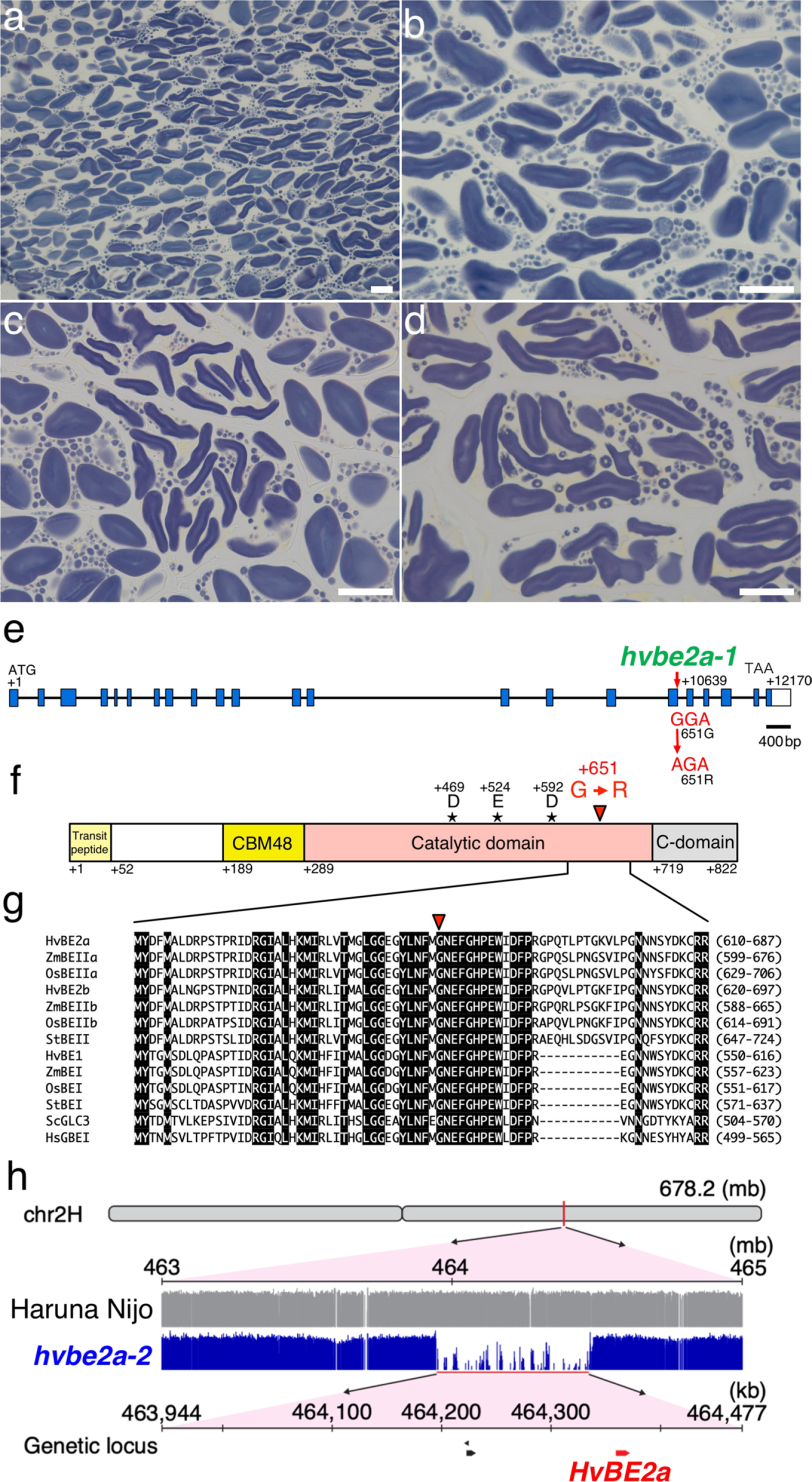
Genetic lesions in *hvbe2a-1* and *hvbe2a-2.* **a**–**b** Iodine-stained thin sections of endosperm cells of F_1_ grains from a cross between *hvbe2a-1* and *hvbe2a-2*. Bars = 20 μm. **c**–**d** Iodine-stained thin sections of *hvbe2a-1* and *hvbe2a-1* mutants, respectively. Bars = 20 μm. **e** The structure of the *HvBE2a* gene on chr2H:464,360,851..464373337 in Haruna_Nijo_pseudomolecules_v1. The coding and untranslated regions are depicted as blue and white boxes, respectively. Introns are indicated by black lines. The exon–intron structure is based on the reported full-length cDNA (AF064560). The adenine in the translation start codon (ATG) is designated as + 1. *hvbe2-1* has a base pair change from G to A at + 10,639, leading to an amino acid substitution of Gly651 by arginine (R). **f** The protein structure of HvBE2a. The first methionine is designated as + 1. The predicted plastidial transit peptide, carbohydrate-binding module of family 48 (CBM48), the central (β/α)_8_ catalytic module of α-amylase family (Catalytic domain) and β-domains typically found in the C terminus of α-amylases family members (C-domain) are depicted according to Pfister and Zeeman (2016) and Noguchi et al. (2011). The putative catalytic triad Asp469-Glu524-Asp592 is shown asterisks. **g** Alignment of sequence around *hvbe2-1* mutation with other starch- and glycogen-branching enzymes. HvBE2a, HvBE2b, HvBE1 (HORVU.MOREX.r3.2HG0165780.1, HORVU.MOREX.r3.2HG0170370.1 and HORVU.MOREX.r3.7HG0751660.1), ZmBEIIa, ZmBEIIb and ZmBEI (Zea mays, AAB67316, NP_001105316, and NP_001105370), OsBEIIa, OsBEIIb and OsBEI (*Oryza sativa*, AB023498, Os02t0528200-01 and Os06t0726400-01), StBEII and StBEI (*Solanum tuberosum*, CAB40748 and CAA49463), ScGLC3 (*Saccharomyces cerevisiae*, AAA34632), HsGBEI (*Homo sapiens*, NM_000158). Perfectly conserved residues are shown in black. Red arrowheads indicate the residues substituted by *hvbe2a-1* mutation. The Clustal W program was used for the alignment. **h** The deletion on chromosome 2H in *hvbe2a-2* including the *HvBE2a* locus. Gray and blue bars represent the relative mapping depth of the NGS short reads for the Haruna Nijo and *hvbe2a-2* genomes, respectively, depicted on a logarithmic scale. The relative positions of three annotated genetic loci, including *HvBE2a*, are marked within the deletion region spanning from 463,944 to 464,477 kb on chromosome 2H

The F1﻿  plant from the cross between *hvbe2a-1* and *hvbe2a-2* exhibited elongated SGs just like *hvbe2a-1* and *hvbe2a-2* (Fig. 2a–d). This means that *hvbe2a-1* and *hvbe2a-2* are allelic to each other. The previous study by Regina (2010) revealed that barley transgenic lines with RNAi-suppressed *HvBE2a* genes exhibited the occasional alternations of SGs in morphology in endosperm. Furthermore, the RNAi lines showed a decrease in the distribution of glucan chain length around DP10 and an increase around DP15 compared to the parental line (Regina et al. 2010). This pattern closely resembled the profile observed in *hvbe2a* mutants in Fig. 1r. The phenotypic similarities encouraged us to determine the sequence of the *HvBE2a* gene in *hvbe2a* mutants. We amplified the genomic region (chr2H:464,360,851–464,373,337 in Haruna Nijo pseudomolecules v1.) covering the *HvBE2a* cDNA sequence (AF064560) from *hvbe2a*-*1* and determined the sequence. In *hvbe2a*-*1*, the guanine residue located 10,639 bp downstream of the first ATG was replaced by adenine (Fig. 2e). The predicted HvBE2a protein has a plastidial transit peptide at its N-terminus, a CBM48, and a central (β/α)_8_ catalytic module characteristic of the α-amylase family (Catalytic domain). Additionally, it features β-domains typically found in the C-terminus of α-amylase family members (C-domain) (Fig. 2f). The *hvbe2a-1* mutation replaces the glycine residue at position 651with an arginine residue. The glycine residue is conserved across various starch- and glycogen-branching enzymes (Fig. 2g). We designed derived cleaved-amplified polymorphic sequence (dCAPS) primers to detect the base change in *hvbe2a-1*^+/–^. The dCAPS primers successfully genotyped the wild-type Haruna Nijo, heterozygous *HvBE2a/hvbe2a-1*, and homozygous mutation of *hvbe2a-1* (Supplementary Fig. 3a). To confirm whether the base change in the *hvbe2a-1* mutant co-segregates with the phenotype of the elongated SGs existence in endosperm, we crossed *hvbe2a-1* with Haruna Nijo and produced F2 populations. Out of the 70 F2 grains, 19 developed elongated SGs in the endosperm, suggesting that the phenotype segregated in a single recessive manner (χ^2^ = 0.17, p = 0.68). Of these 19 grains, fifteen were randomly selected and grown into seedlings for genotyping. The genotyping using the dCAPS primer showed that all the 15 plants were homozygous for the *hvbe2a-1* base change (Supplementary Fig. 3b). This result supports the idea that the base change in the *HvBEIIa* gene of the *hvbe2a-1* mutant is responsible for the elongated SG phenotype.

Next-generation sequencing of the *hvbe2a-2* mutant genome, followed by the mapping of the obtained short reads against the Haruna Nijo genome, identified the genomic region with missing reads in *hvbe2a-2* (Fig. 2h). This region, extending from 464,944 to 464,477 kb on chromosome 2H, includes the *HvBE2a* gene and two other annotated genes. Thus, *hvbe2a-2* is missing the *HvBE2a* gene due to a deletion.

### HvBE2a protein accumulation and branching enzyme activity in *hvbe2a* mutants

**Fig. 3:**
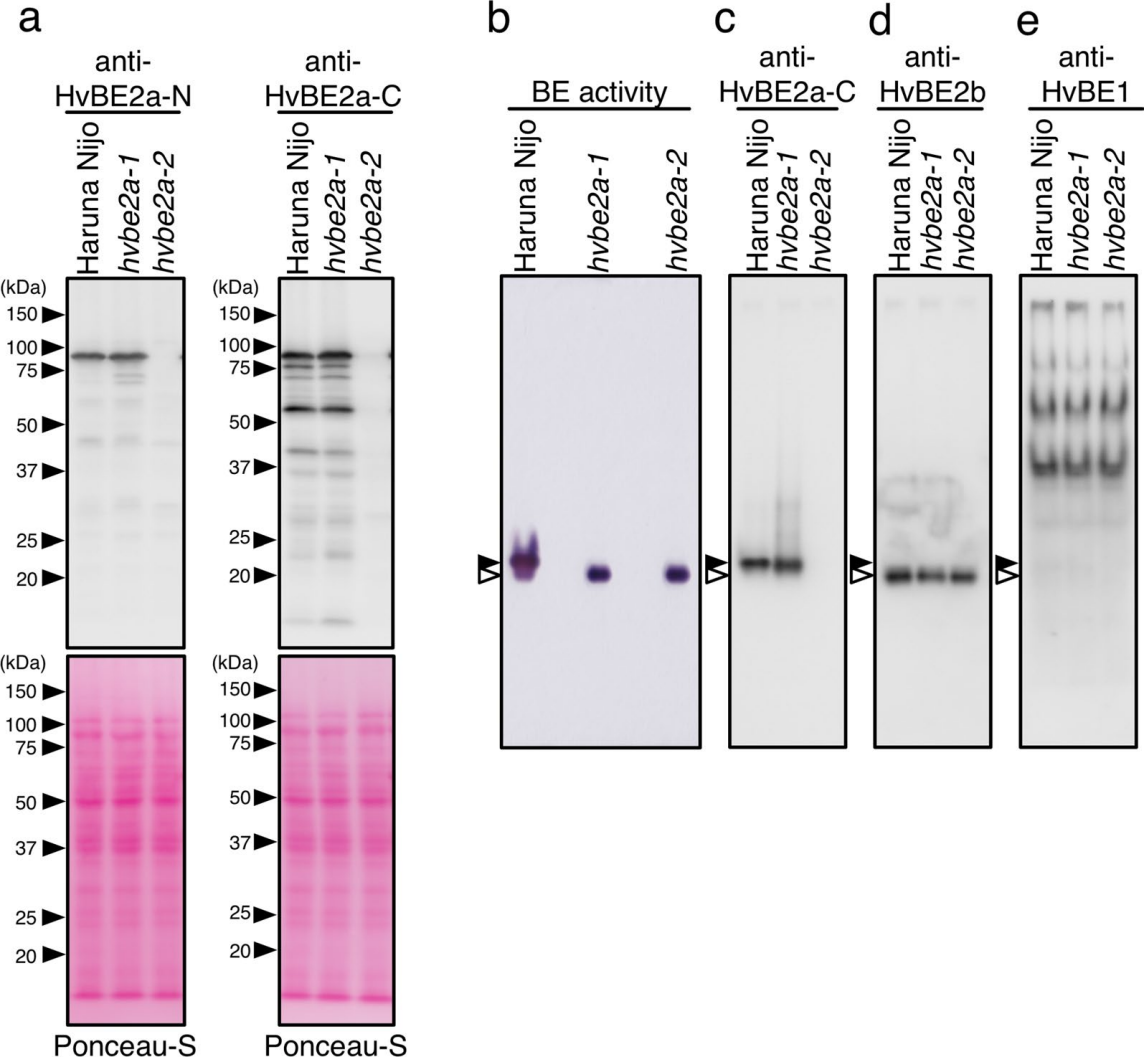
Starch branching enzyme activity and protein accumulation in *hvbe2a* mutants. **a** Immunoblot analysis with anti-HvBE2a-N and anti-HvBE2a-C antibodies after SDS-PAGE of developing endosperm extracts from Haruna Nijo, *hvbe2a-1* and *hvbe2a-2*. The molecular masses are given on the left in kDa. Membranes are stained with Ponceau-S to verify equal protein loading and transfer. **b** Native-PAGE activity staining of starch branching enzymes in mutants and immunoblot analysis after Native-PAGE. Proteins from at 14 days after awn emergence (7.5 μg) were loaded on each lane. **c**–**e** Immunoblot analysis using anti-HvBE2a-C, anti-HvBE2b and anti-HvBE1, respectively. Closed and open arrowheads indicate the position of HvBE2a and HvBE2b, respectively

Next, we examined the accumulation of the HvBE2a protein and its enzyme activity in *hvbe2a* mutants. The amino acid sequences of the synthetic peptides used to create the antibodies are shown in Supplemental Fig. 4. The two anti-HvBE2a antibodies recognize the N-terminus and C-terminus of the mature HvBE2a sequences without the transit peptide, respectively. We refer to the two antibodies as anti-HvBE2a-N and anti-HvBE2a-C. Both antibodies recognized the band around 90 kDa in Haruna Nijo and *hvbe2a-1*, but not in *hvbe2a-2* mutant in the immunoblot analysis of the developing grains (Fig. 3a). The band size is consistent with the expected molecular weight of HvBE2a, which is 87.6 kDa, excluding the transit peptide. The absence of the detected band in the *hvbe2a-2* is due to the deletion of the *HvBE2a* gene (Fig. 2h). In both Haruna Nijo and *hvbe2a-1*, anti-HvBE2a-C antibody detected the smaller-sized bands together with the 90-kDa band, which could be degradation products of HvBE2a since they were absent in *hvbe2a-2*. Out of the degradation products, the band around 60 kDa, detected with anti-HvBE2a-C antibody, showed significant intensity comparable to that of the full-length band of HvBE2a, implying that it is the most abundant degradation product. This band was not detected with anti-HvBE2a-N antibody, suggesting that the N-terminus is missing in this degradation product. This suggests that HvBE2a is more prone to degradation from the N-terminus.

Next, we investigated BE activity in the maturing endosperm of *hvbe2a* mutants. In barley, besides HvBE2a, there are other BE isozymes, namely HvBE2b and HvBE1. These show 76% and 46% amino acid sequence identity with HvBE2a, respectively. To discern their individual activities, we utilized an in-gel Native-PAGE activity assay, which separates these isozymes on the gel. In Haruna Nijo maturing endosperm at 14 DAA, two bands with BE activity were detected with slightly different mobilities on the gel (Fig. 3b). The upper band was major and the lower band was minor (Fig. 3b). In the case of *hvbe2a-1* and *hvbe2a-2* mutants, the upper band was not detected, while the only lower band were detected to have BE activity. To ascertain the precise locations of the bands corresponding to each isozyme in Native-PAGE, we constructed specific antibodies recognizing HvBE2b and HvBE1, respectively in addition to HvBE2a (Supplemental Fig. 4). Immunoblot analysis following Native-PAGE revealed that the upper and lower bands with BE activities in Haruna Nijo corresponded to HvBE2a and HvBE2b, respectively (Fig. 3c, d). This suggests that HvBE2a is the major BE enzyme in the developing endosperm of Haruna Nijo. The protein accumulation level of HvBE2a in *hvbe2a-1* was almost the same as in Haruna Nijo. The absence of BE activity from HvBE2a in *hvbe2a-1* indicates that the amino acid substitution in *hvbe2a-1* does not impact protein accumulation but is crucial for enzymatic activity of HvBE2a. Although HvBE1 was detected as more than two bands with different mobilities on the immunoblotted membrane, no BE activity was observed at these positions under our experimental conditions (Fig. 3b, e).

**Fig. 4:**
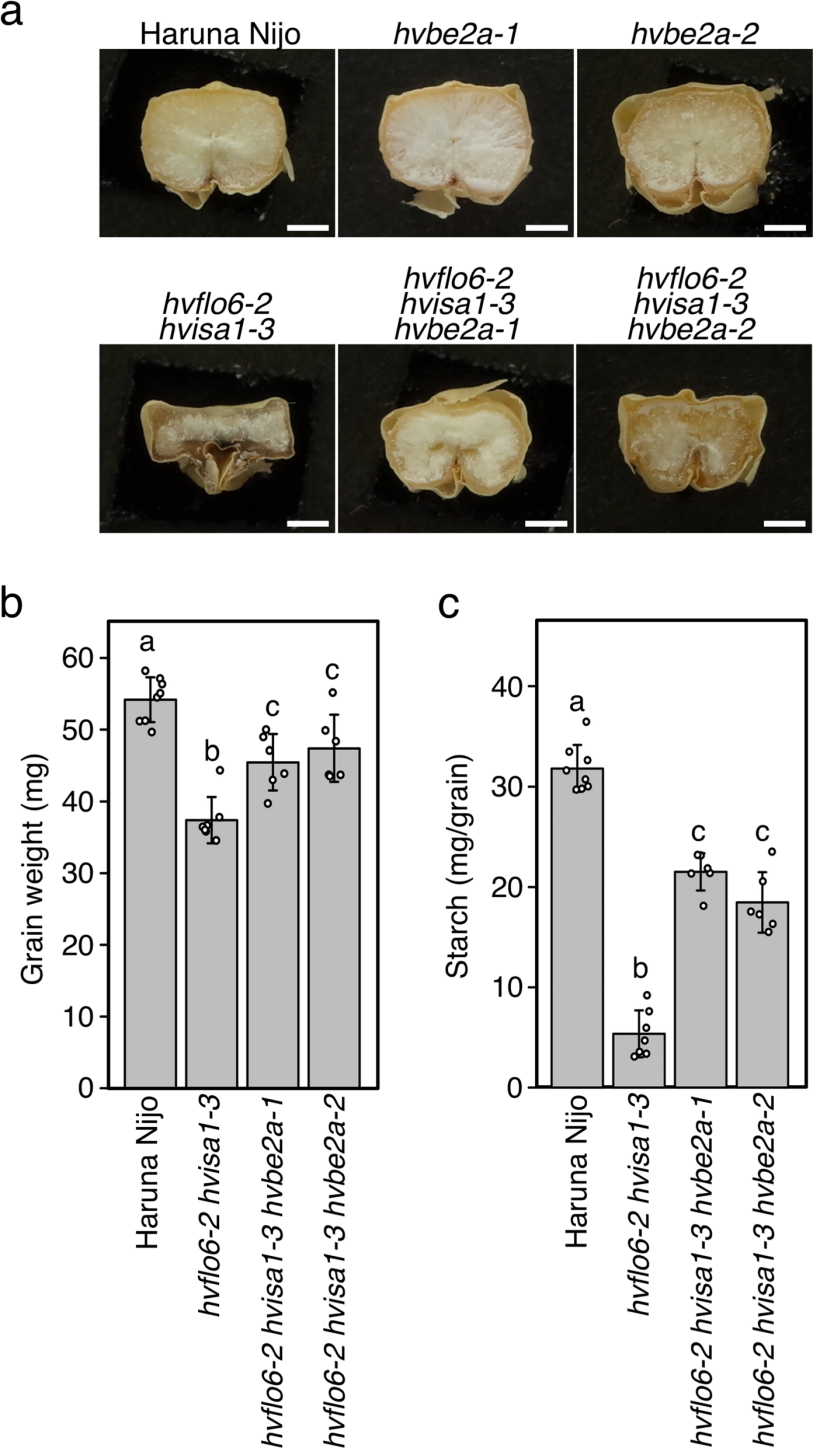
Suppressive effect of *hvbe2a* mutations against starchless phenotype of *hvflo6-2 hvisa1-3* grain*.* **a** Cross-sections of a mature grain of Haruna Nijo and mutants. Bars = 1 mm. **b** Single grain weight of triple mutant mature grains (*n* = 6–8). **c** Starch amount per grain of triple mutant mature grains (*n* = 6–8). Data are given as means ± SD. Statistical comparisons were performed using Tukey’s HSD. The same letters above the bars represent statistically indistinguishable groups, and different letters represent statistically different groups (*p* < 0.05)

### *hvbe2a* suppresses the starchless phenotype of *hvflo6 hvisa1*

The *hvflo6* mutation enhances the *hvisa1* phenotype, leading to a significant reduction in starch content in grains and severe grain shrinkage in *hvflo6 hvisa1* double mutants (Matsushima et al. 2023). In rice, the *isa1* phenotype is mitigated by the mutations of *be2a* and *be2b* (Lee et al. 2017; Nagamatsu et al. 2022). We therefore generated the *hvflo6 hvisa1 hvbe2a* triple mutant to examine the suppressive effect of *hvbe2a* mutations against the *hvflo6 hvisa1* phenotypes. The *hvbe2a-1* and *hvbe2a-2* mutants did not show any significant differences in grain cross-sections compared to Haruna Nijo (Fig. 4a). The *hvflo6-2 hvisa1-3* grains were shrunken. Interestingly, when *hvbe2a-1* and *hvbe2a-2* mutations were introduced into the *hvflo6-2 hvisa1-3* background, the grain shrinkage phenotype was partially rescued in both triple mutants (Fig. 4a). The results show that *hvbe2a* mutations have a suppressive effect on the *hvflo6-2 hvisa1-3* phenotype. We also measured the grain weight and starch content in the grains of the triple mutants (Fig. 4b, c). For the triple mutants, the individual grain weight was higher than that of the *hvflo6-2 hvisa1-3* double mutant but lower than that of Haruna Nijo (Fig. 4b). Similarly, the starch content of the grain in triple mutants was higher than that of the *hvflo6-2 hvisa1-3* grain but lower than that of Haruna Nijo (Fig. 4c). This indicates that loss of HvBE2a function did not completely suppress the double mutant phenotype of *hvflo6-2 hvisa1-3*.

**Fig. 5:**
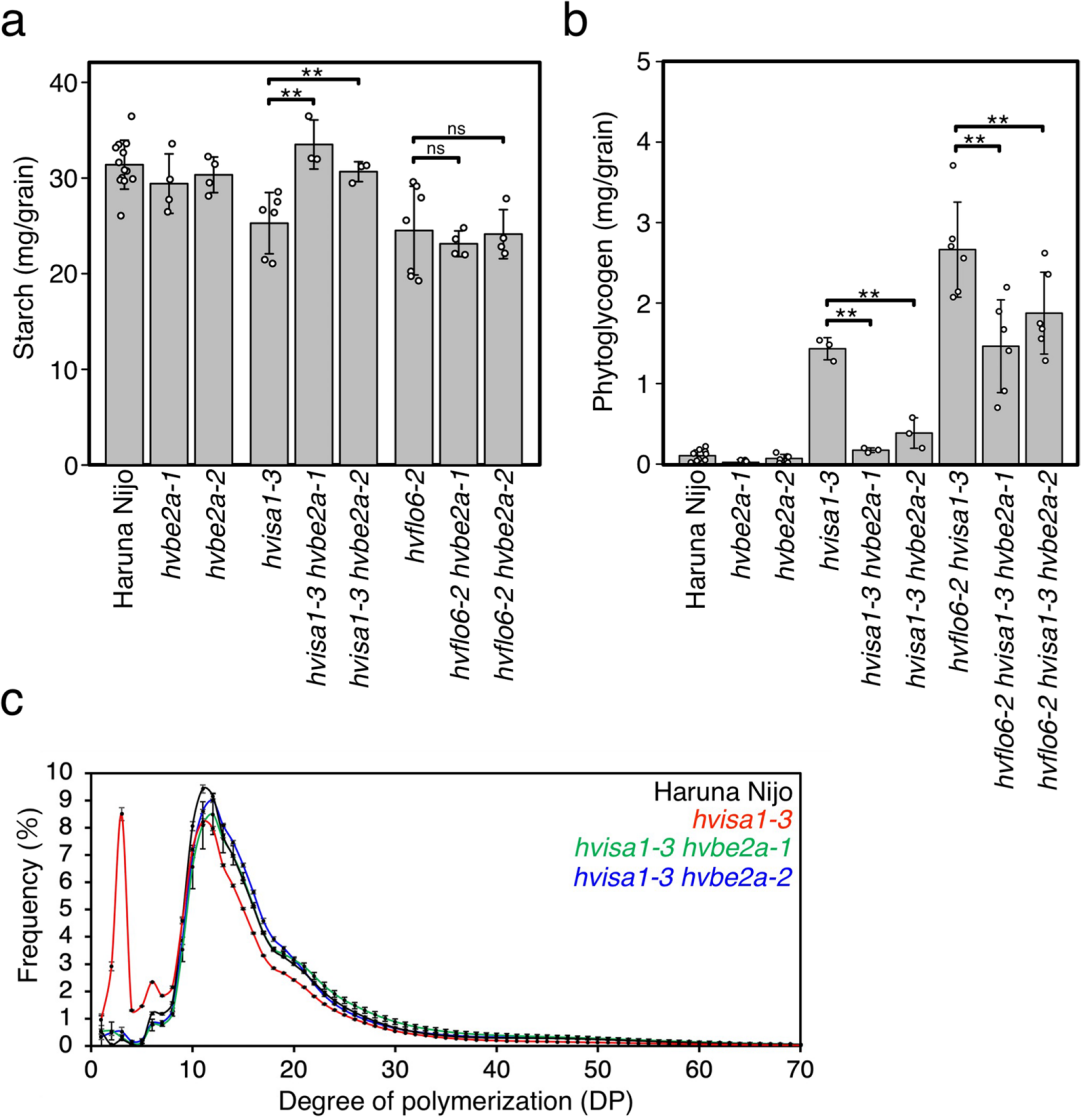
Suppressive effect of *hvbe2a* mutations against *hvisa1-3* starch properties. **a** Starch amount per grain of single and double mutants. Data are given as means ± SD using at least three biological replicates. Statistical comparisons were performed using Welch’s *t*-test (**, *p* < 0.01; and ns, not significant at *p* = 0.05). The starch reduction in *hvisa1-3* was recovered by adding *hvbe2a* mutations. In contrast, the starch reduction in *hvflo6-2* was not affected by *hvbe2a* mutations. **b** Amount of phytoglycogen per mature grain in double and triple mutants. Data are given as means ± SD using at least three biological replicates. Statistical comparisons were performed using Welch’s *t*-test (**, *p* < 0.01). The increases of phytoglycogen in *hvisa1-3* and *hvflo6-2 hvisa1-3* were suppressed by adding *hvbe2a* mutations. **c** Glucan chain-length distribution of α-glucans of mature grains. Haruna Nijo, *hvisa1-3*, *hvisa1-3 hvbe2a-1, hvisa1-3 hvbe2a-2* are indicated by the black, red, green and blue lines, respectively. The values for *hvisa1-3* are identical to the data in Matsushima et al. (2023). Data are given as means ± SD. All data were obtained from at least three independent grains

To confirm which mutations, *hvflo6-2* or *hvisa1-3*, are targeted by the suppressive effect of *hvbe2a*, we generated a series of double mutants, including *hvisa1-3 hvbe2a-1, hvisa1-3 hvbe2a-2*, *hvflo6-2 hvbe2a-1* and *hvflo6-2 hvbe2a-2*. In line with the previous observation (Matsushima et al. 2023), the starch content of the single mutants of *hvflo6-2* and *hvisa1-3* grains was reduced compared to Haruna Nijo (Fig. 5a). The starch content was higher in the mutants of *hvisa1-3 hvbe2a-1* and *hvisa1-3 hvbe2a-2* compared to the *hvisa1-3* single mutant. Their starch content was restored to the wild-type level (Fig. 5a). In contrast, there was no significant difference in the starch content of *hvflo6-2 hvbe2a-1* and *hvflo6-2 hvbe2a-2* grains compared to the *hvflo6-2* single mutant (Fig. 5a). This result means that the suppressive effect of the *hvbe2a* mutations predominantly targets *hvisa1-3*, rather than *hvflo6-2*.

We have previously shown that the *hvisa1-3* grains accumulate significantly more phytoglycogen compared to the wild type (Matsushima et al. 2023). Furthermore, this accumulation was found to be even more pronounced with the addition of the *hvflo6-2* mutation. To determine whether the *hvbe2a* mutations suppress the phytoglycogen accumulation of *hvisa1-3*, we measured the phytoglycogen accumulation in *hvisa1-3 hvbe2a* double mutants. The amount of phytoglycogen in *hvisa-3 hvbe2a-1* and *hvisa-3 hvbe2a-2* mutants decreased by 88% and 77%, respectively, compared to *hvisa-3* (Fig. 5b). These data demonstrate a noteworthy decrease in phytoglycogen accumulation in both *hvisa1-3 hvbe2a-1* and *hvisa1-3 hvbe2a-2* mutants, compared to the single *hvisa1-3* mutant (Fig. 5b). This result confirms that the suppressive effect of the *hvbe2a* mutations primarily targets *hvisa1*. We also examined the suppressive effect of *hvbe2a* mutations on phytoglycogen accumulation in the *hvflo6-2 hvisa1-3* background. The amount of phytoglycogen in *hvflo6-2 hvisa-3 hvbe2a-1* and *hvflo6-2 hvisa-3 hvbe2a-2* triple mutants decreased by 45% and 30%, respectively, compared to *hvflo6-2 hvisa-3* double mutants (Fig. 5b). In the triple mutants, the phytoglycogen were decreased compared to the *hvflo6-2 hvisa1-3,* but not to the level of *hvisa1-3 hvbe2a-1* and *hvisa1-3 hvbe2a-2* (Fig. 5b). This indicates that *hvflo6-2* and *hvbe2a* are in competition to either enhance or suppress the phenotype of *hvisa1-3*.

The distribution of glucan chain length in α-glucan from *hvisa1-3 hvbe2a-1* and *hvisa1-3 hvbe2a-2* grains closely resembles that of Haruna Nijo (Fig. 5c). While, *hvisa1-3* single mutant exhibits a higher abundance of shorter glucose chains (DP < 10) and a lower abundance of longer glucose chains (DP > 10) compared to Haruna Nijo. This also supports the suppressive effect of the *hvbe2a* mutations against *hvisa1-3*.

### Transformation of starch granule morphology by *hvisa1* and *hvbe2a*

**Fig. 6:**
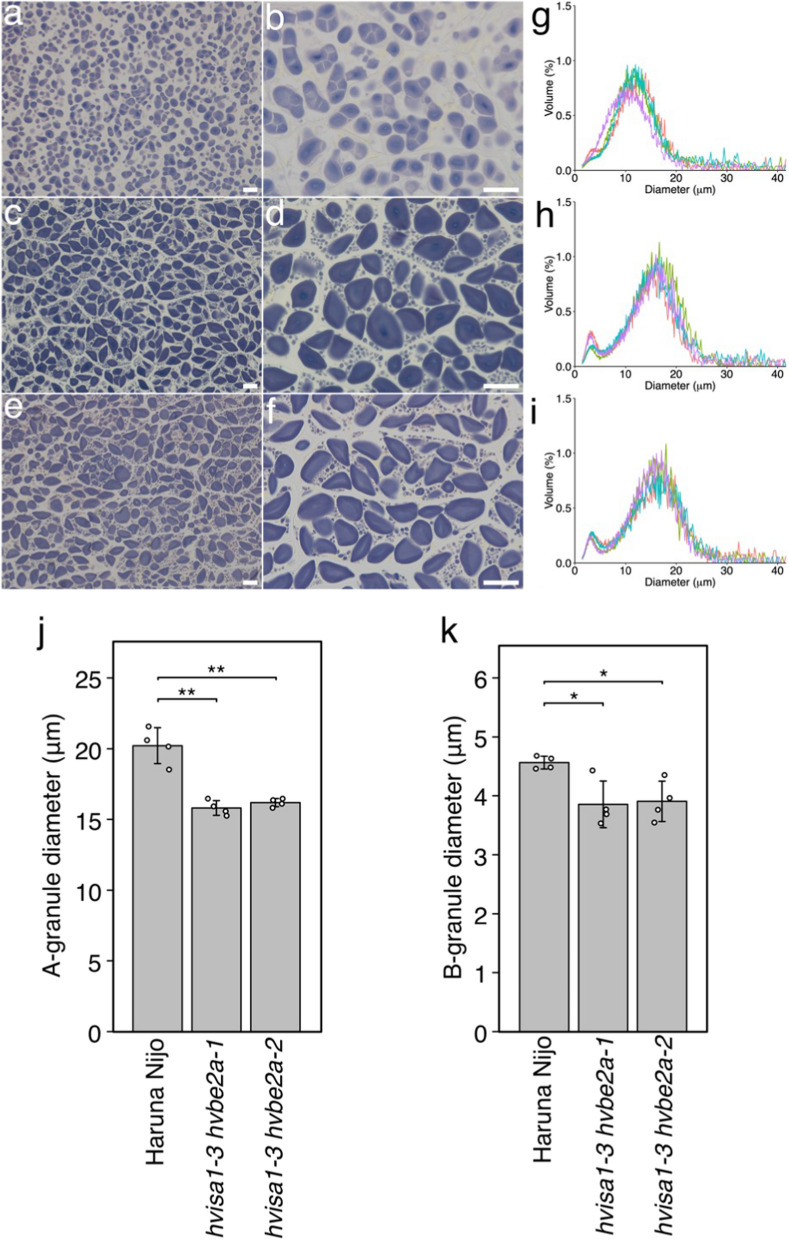
Suppressive effect of *hvbe2a* mutations on *hvisa1-3* starch granule morphology in endosperm. **a**–**f** Iodine-stained thin sections of endosperm cells of *hvisa1-3* (a and b), *hvisa1-3 hvbe2a-1* (c and d), and *hvisa1-3 hvbe2a-2* (e and f). Bars = 20 μm. **g**–**i** Granule size distributions of *hvisa1-3*, *hvisa1-3 hvbe2a-1* and *hvisa1-3 hvbe2a-2*, respectively. The relative percent volume of each diameter was determined using a Coulter Counter. **j**–**k** The average diameter of A- and B-type granules, respectively, extracted from the relative percent volume vs. diameter plots of *hvisa1-3 hvbe2a-1* and *hvisa1-3 hvbe2a-2* (h and i) by fitting a bimodal mixed normal and lognormal distributions (n = 4). The values for Haruna Nijo are identical to the data presented in Fig. 1 (n, o). Data are given as means ± SD. Statistical comparisons were performed using Tukey’s HSD. The same letters above the bars represent statistically indistinguishable groups, and different letters represent statistically different groups (*p* < 0.05)

**Fig. 7:**
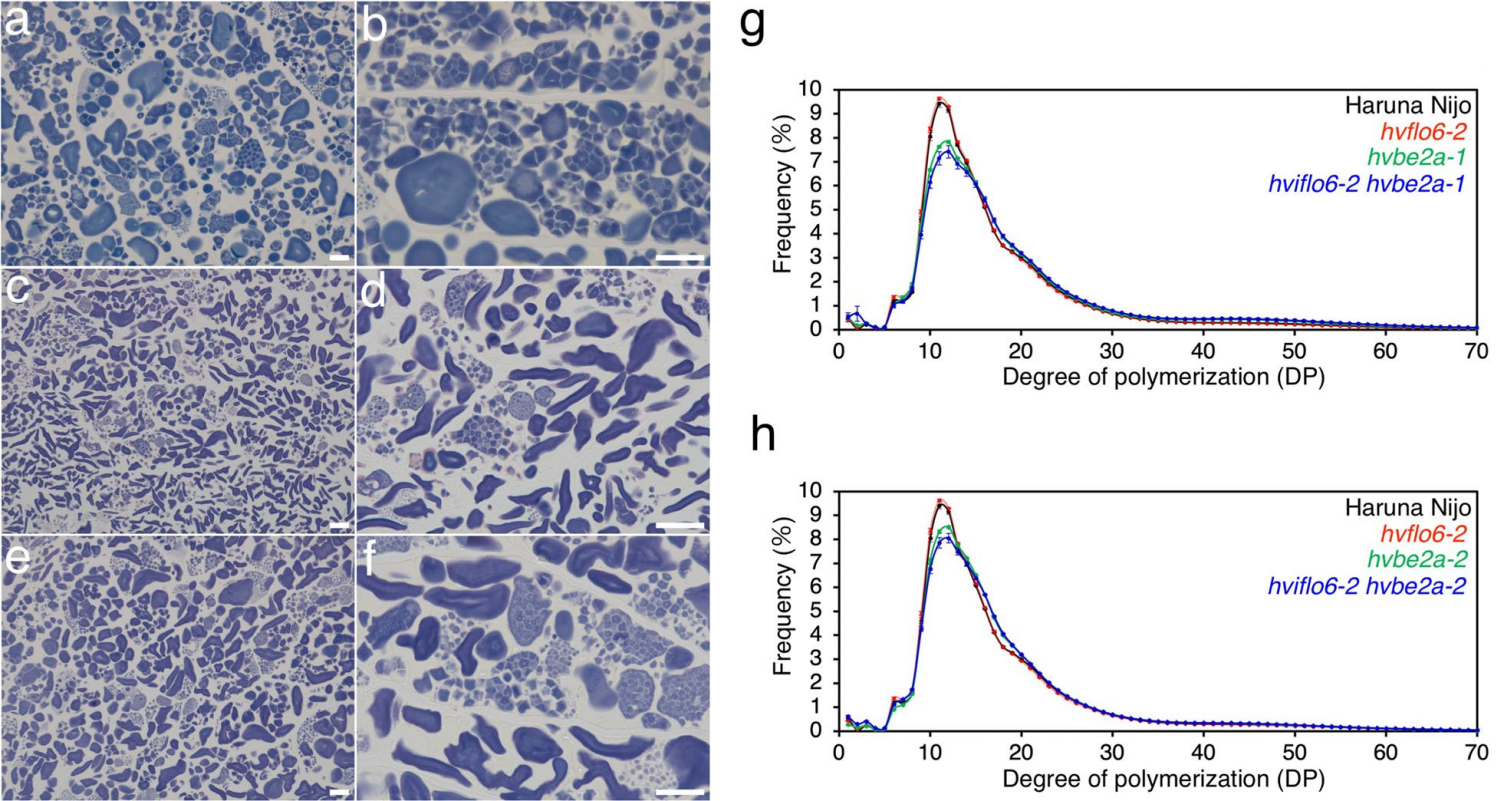
Starch granule morphology and glucan chain-length distribution of *hvflo6 hvbe2a* double mutants. **a**–**f** Iodine-stained thin sections of endosperm cells of *hvflo6-2* (a and b), *hvflo6-2 hvbe2a-1* (c and d), and *hvflo6-2 hvbe2a-2* (e and f). Bars = 20 μm. **g** Glucan chain-length distribution of α-glucans of mature grains. Haruna Nijo, *hvflo6-2, hvbe2a-1, hvflo6-2 hvbe2a-1* are indicated by the black, red, green and blue lines, respectively. **h** Glucan chain-length distribution of α-glucans of mature grains. Haruna Nijo, *hvflo6-2, hvbe2a-2, hvflo6-2 hvbe2a-2* are indicated by the black, red, green and blue lines, respectively. The values for *hvflo6-2* are identical to the data in Matsushima et al. (2023). Data are given as means ± SD. All data were obtained from at least three independent grains

Next, we investigated the impact of *hvbe2a* mutations on the SG morphology of the *hvisa1-3* mutant. In *hvisa1-3*, compound SGs were well developed in the endosperm, replacing the original bimodal simple SGs typical of barley (Fig. 6a, b). This is consistent with previous observations (Matsushima et al. 2023). In the endosperm of *hvisa1-3 hvbe2a-1* and *hvisa1-3 hvbe2a-2*, typical A- and B-type SGs were developed (Fig. 6c–f). When SGs of *hvisa1-3* were purified and analyzed using a Coulter Counter, the granule size distribution with typical bimodal peaks of A- and B-type SGs was not observed (Fig. 6g). Instead, a single peak was predominant. The observed peak is most likely attributed to the starch particles that formed the compound SGs of *hvisa1-3* and subsequently disintegrated during purification. In the case of *hvisa1-3 hvbe2a-1* and *hvisa1-3 hvbe2a-2*, the Coulter Counter analysis consistently showed bimodal peaks (Fig. 6h, i). This suggests that in barley, SGs change from authentic bimodal type to compound type due to the *hvisa1* mutation, and the *hvbe2a* mutation can reverse these compound SGs back to the bimodal type. We have analyzed the average diameters of A- and B-type granules in *hvisa1-3 hvbe2a-1* and *hvisa1-3 hvbe2a-2* mutants. Our findings reveal that for A-type granules, the diameter in both *hvisa1-3 hvbe2a-1* and *hvisa1-3 hvbe2a-2* mutants was approximately 80% of that in Haruna Nijo (Fig. 6j). Similarly, for B-type granules, the diameter in both mutants was around 85% relative to that in Haruna Nijo (Fig. 6k). The observation of smaller bimodal SGs in the *hvisa1-3 hvbe2a* mutants, compared to those in Haruna Nijo, indicates that the depletion of HvBE2a does not fully suppress the SG morphological changes induced by *hvisa1-3*. We also investigated the impact of the *hvbe2a* mutations on SG morphology in the *hvflo6-2* mutant. In *hvflo6-2*, compound SGs were well developed in the endosperm (Fig. 7a, b), consistent with previous observations (Matsushima et al. 2023). In the endosperm of *hvflo6-2 hvbe2a-1* and *hvflo6-2 hvbe2a-2*, *hvbe2a*-specific elongated SGs were clearly observed alongside the *hvflo6-2*-specific compound SGs (Fig. 7c–f). This suggests that the effects of the *hvflo6-2* and *hvbe2a* mutations are cumulative with respect to SG morphology. Regarding the α-glucan chain length distribution, *hvflo6-2* exhibited a distribution nearly identical to that of Haruna Nijo (Fig. 7g, h), consistent with previous research (Matsushima et al. 2023). When comparing the *hvbe2a-1* mutant with the *hvflo6-2 hvbe2a-1* double mutant, there was a slight reduction in glucose chains ranging from DP7 to DP15 in the double mutant (Fig. 7g). Similarly, the *hvflo6-2 hvbe2a-2* double mutant showed a slight reduction in glucose chains from DP10 to DP15 compared to the *hvbe2a-2* mutant (Fig. 7h). However, no other significant differences in glucose chain length were observed between the double and single mutants (Fig. 7g, h). These findings indicate that the genetic interaction between the *hvflo6* and *hvbe2a* mutations differs from the suppressive interaction observed between the *hvisa1* and *hvbe2a* mutations.

### Absence of suppressive effect of *hvbe2a* against *hvflo6 hvisa1* in pollen

**Fig. 8:**
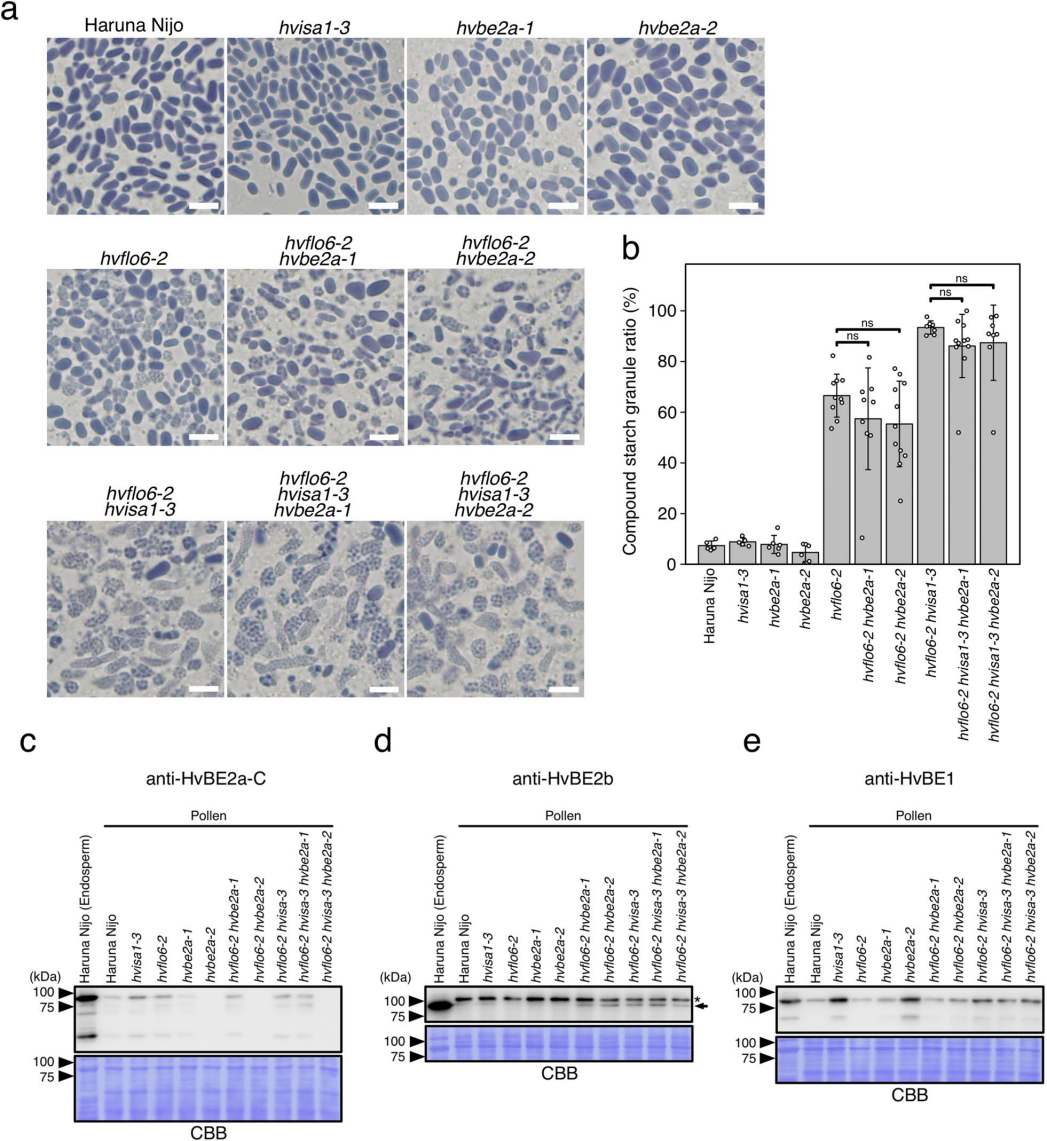
Starch granules in mutant pollen. **a** Iodine-stained SGs released from squashed pollen grains. Bars = 5 μm. **b** The percentage of starch granules in compound form, as determined from microscopic images. Data are given as means ± SD. Statistical comparisons were performed using Welch’s *t*-test (ns, not significant at *p* = 0.05). **c**–**e** Accumulation of HvBE2a, HvBE2b, and HvBE1 in pollen. Protein extracts from developing endosperm at 14 days after awn emergence (DAA) and mature pollen at 3 DAA from Haruna Nijo and mutants were subjected to SDS-PAGE followed by the immunoblot analysis using anti-HvBE2a-C, anti-HvBE2b, and anti-HvBE1 antibodies. Each lane contains 5 μg of protein. The molecular masses are indicated on the left in kDa. Membranes are stained with CBB to verify equal protein loading and transfer. Arrow and asterisk in **d** indicate the positions of specific and non-specific bands, respectively

Pollen, as well as endosperm, accumulates storage starch. The rod-shaped SGs were well developed in pollen from Haruna Nijo, *hvisa1-3, hvbe2a-1* and *hvbe2a-2* (Fig. 8a). In the *hvflo6-2* mutant, a notable increase in compound SGs was observed in addition to the rod-shaped SGs. The proportion of the compound SGs in *hvflo6-2* pollen was up to 66.5%, whereas those in Haruna Nijo, *hvisa1-3*, *hvbe2a-1*, and *hvbe2a-2* were less than 9% (Fig. 8b). The proportion of the compound SGs in the *hvisa1-3 hvbe2a-1* and *hvisa1-3 hvbe2a-1* pollens were not statistically significant compared to *hvisa1-3* pollen (Supplemental Fig. 5a–b). In contrast, the double mutants, *hvflo6-2 hvbe2a-1* and *hvflo6-2 hvbe2a-2,* exhibited well-developed compound SGs at the same level as *hvflo6-2* (Fig. 8a, b). This indicates that *hvbe2a* does not suppress *hvflo6-2* in terms of compound SG formation in pollen. In contrast, the *hvisa1-3* mutation significantly enhanced the formation of compound SGs in *hvflo6-2* pollen (Fig. 8a). In the *hvflo6-2 hvisa1-3* pollen, more than 90% of SGs were of the compound type (Fig. 8b). This implies that HvISA1 is involved in compensating for HvFLO6 function in pollen, especially in the *hvflo6-2* background.　We also observed pollen SGs in triple mutants, *hvflo6-2 hvisa1-3 hvbe2-1* and *hvflo6-2 hvisa1-3 hvbe2-2*. The proportion of compound SGs in these triple mutants did not significantly differ from that in the *hvflo6-2 hvisa1-3* mutants (Fig. 8b). This observation supports the hypothesis that the *hvbe2a* mutation cannot suppress the formation of compound SGs induced by *hvflo6-2* or *hvisa1-3* in pollen.

To investigate the protein accumulation of HvBE2a, HvBE2b, and HvBE1 in pollen, pollen protein extracts were subjected to the immunoblot analysis (Fig. 8c–e). HvBE2a accumulation was not detected in the mutants’ pollen harboring *hvbe2a-2* allele (*hvbe2a-2*, *hvflo6-2 hvbe2a-2* and *hvflo6-2 hvisa1-3 hvbe2a-2*) because of the deletion of the *HvBE2a* gene (Fig. 8c). In pollen, HvBE2a accumulation is lower overall, even in Haruna Nijo, when compared to the endosperm (Fig. 8c). The HvBE2a band intensity of the Haruna Nijo pollen is slightly stronger compared to that of *hvbe2a-1* pollen (Fig. 8c). This may indicate that the amino acid substitution in *hvbe2a-1* affects protein accumulation particularly in pollen. In the *hvisa-1-3* and *hvflo6-2* mutants, the accumulation of HvBE2a was slightly higher than in Haruna Nijo.

Anti-HvBE2b antibodies recognized the band around 90 kDa in the developing endosperm (Fig. 8d, arrow). The band size is consistent with the expected molecular weight of HvBE2b (87.9 kDa). The bands with the same size were detected in pollen of *hvflo6-2 hvbe2a-1*, *hvflo6-2 hvbe2a-2*, *hvflo6-2 hvisa1-3*, *hvflo6-2 hvisa1-3 hvbe2a-1*, and *hvflo6-2 hvisa1-3 hvbe2a-2*. It is noted that these mutants are characterized by the increased proportion of compound SGs in their pollen (Fig. 8b). Anti-HvBE2b antibodies also detected bands around 100 kDa only in the pollen samples (Fig. 8d, asterisk). These bands are significantly larger than the expected molecular weight of HvBE2b, and may be non-specific bands unique to the pollen samples. Anti-HvBE1 antibodies recognized the band around 90 kDa in developing endosperm of Haruna Nijo, which is consistent with the expected molecular weight of HvBE1 (86.5 kDa) (Fig. 8e). In *hvisa-1-3* and *hvbe2a-2* pollen*,* HvBE1 was significantly accumulated compared to Haruna Nijo.

### Fertility of pollen with compound starch granules

In order to evaluate the fertility of *hvflo6-2* pollen with compound SGs, we studied the SGs in mature pollens from heterozygous plant (*HvFLO6*/*hvflo6-2*). We measured the segregation ratio of *hvflo6-2* mutant pollen (having compound SGs) to wild-type pollen (containing normal rod-shaped simple SGs) (Table 1). The segregation ratio was almost 1:1. This suggests that the *hvflo6-2* mutation behaves in a gametophytic manner in pollen and that *hvflo6-2* pollen matures successfully without aborting during the developmental stage. We then obtained self-fertilized F2 grains from *HvFLO6*/*hvflo6-2* heterozygous plants and examined the segregation ratio of *hvflo6-2* grains to wild-type grains by observing SG morphology in each grain (Table 2). The segregation ratio was 1:3 without any segregation distortion. This indicates that the fertility of *hvflo6-2* pollen is not significantly different from that of wild-type pollen. These results suggest that the increase in compound SGs in *hvflo6-2* pollen does not significantly affect the pollen fertility.

**Table 1:** Segregation of *hvflo6* pollen of F1 plant

Parental genotype	No. of *hvflo6* pollen	No. of wild-type pollen	Total	*X*^2^ value (*p* value) for 1:1 Segregation
*HvFLO6/hvflo6-2*	126	147	273	1.6 (0.2)

Mature anthers from the F1 hybrid between *hvflo6-2* and wild-type cv Haruna Nijo were disrupted with forceps in dilute Lugol solution on a glass slide to obtain iodine-stained pollens. The released pollen were subsequently examined with the microscope

**Table 2:** Segregation of *hvflo6-2* seeds of the F2 population

Parental genotype	No. of *hvflo6* seeds	No. of wild-type seeds	Total	*X*^2^ value (*p* value) for 1:3 segregation
*HvFLO6/hvflo6-2*	10	30	40	0 (1)

F2 seeds were obtained from the cross between *hvflo6-2* and wild-type cv Haruna Nijo. Endosperm thin sections were prepared from 40 F2 seeds. Starch granules were examined with the microscope for phenotyping

In our previous paper (Matsushima et al. 2023), we presented the co-segregation analysis of shrunken grains caused by the *hvflo6-2 hvisa1-3* genotype. This analysis used the selfed population of *HvFLO6*/*hvflo6-2 hvisa1-3* plants, where *hvflo6-2* is heterozygous, and *hvisa1-3* is homozygous. In the selfed population, the segregation ratio of shrunken grains to normal grains was approximately 1:3 (Matsushima et al. 2023). Genotyping of the grains revealed that all shrunken grains were consistent with the *hvflo6-2 hvisa1-3* double homozygous genotype. On the other hand, normal grains were either *HvFLO6*/*hvflo6-2 hvisa1-3* or *HvFLO6 hvisa1-3*. This indicates that the *hvflo6-2* mutation segregates as a single recessive allele in the *hvisa1-3* background. This undistorted co-segregation experiment suggests that the fertility of *hvflo6-2 hvisa1-3* pollen (having compound SGs) is not significantly different from that of *hvisa1-3* pollen (containing normal rod-shaped simple SGs).

## Discussion

### Isolation of mutants defective in *HvBE2a* gene

BE is an enzyme that creates branches during amylopectin synthesis (Nakamura 2002; Pfister and Zeeman 2016). In barley and wheat endosperms, BE2a is the major BE isozyme in endosperm (Regina et al. 2006, 2010). In this study, two barley mutants with genetic lesions in *HvBE2a* gene were isolated through screening based on the SG morphology (Fig. 1). In *hvbe2a-1* and *hvbe2a-2* mutants, elongated SGs were observed in the endosperm cells (Fig. 1g–j). Previously, transgenic barley with suppressed *HvBE2a* expression using RNAi were created, but mutants have not been isolated (Regina et al. 2010). The *hvbe2a-1* mutant had a nucleotide change causing an amino acid substitution, and the *hvbe2a-2* mutant had the complete deletion of the *HvBE2a* gene (Fig. 2). In the *hvbe2a-1* mutant, the HvBE2a-1 protein accumulated in similar amounts to the wild-type HvBE2a protein in Haruna Nijo. However, no BE activity was detected from the HvBE2a-1 protein (Fig. 3b, c). Therefore, the substituted amino acid residue in the HvBE2a-1 protein does not affect protein accumulation but is crucial for the enzymatic activity. This observation aligns with the location of the amino acid substitution near the catalytic triad, and the fact that this residue is conserved among BEs of various plant species as well as glycogen BEs from animals and bacteria (Fig. 2g).

BEIIb is the primary BE in rice endosperm (Mizuno et al. 1993). Rice *beIIb* mutant *EM10* fails to accumulate BEIIb protein, while another *beIIb* mutant, *ssg3*, carries a base substitution leading to an amino acid change in BEIIb (Mizuno et al. 1993; Matsushima et al. 2010). *ssg3* mutation allows the accumulation of BEIIb protein but eliminates its enzymatic activity. *EM10* and *ssg3* exhibit similar phenotypes, characterized by the reduced polygonal starch particles and altered glucan chain length distributions (Matsushima et al. 2010; Nagamatsu et al. 2022). However, they differ in starch biosynthetic enzyme complex formation (Crofts et al. 2018). This difference is thought to arise from the inactive enzyme still having the ability to contribute to complex formation (Crofts et al. 2018). In this study, in the glucan chain length distribution, *hvbe2a-1* exhibited a more pronounced reduction around DP11 compared to *hvbe2a-2* (Fig. 1q, r). The difference may be due to the distinct protein complexes of starch biosynthetic enzymes. In the *hvbe2a-1* mutant, the HvBE2a protein is present without biochemical activity, which may prevent other isozymes from replacing it. This could lead to a more severe phenotype compared to the complete absence of HvBE2a in *hvbe2a-2*. However, we did not observe any morphological differences in SGs between *hvbe2a-1* and *hvbe2a-2* (Fig. 1g–j). This is because both mutants have a substantial number of normal SGs along with elongated ones, making it difficult to distinguish differences in SG morphology between *hvbe2a* mutants.

### Suppressive impact of *hvbe2a* against *hvisa1* in endosperm

In *hvisa1-3* grains, there was a reduction in total starch and an increase in phytoglycogen. However, these phenotypes were less pronounced in the *hvbe2a-1* and *hvbe2a-2* background. (Fig. 5a, b). This suggests that the *hvbe2a-1* and *hvbe2a-2* mutations are epistatic to the *hvisa1-3* mutation. BEs are essential for catalyzing glucose chain branching during amylopectin biosynthesis. However, BEs can sometimes lead to branching at inappropriate positions. In contrast, ISA1 effectively removes glucose chains attached to inappropriate positions on amylopectin to avoid excessive branching caused by BEs (Nakamura 2002; Smith and Zeeman 2020). The absence of HvBE2a, a major BE in barley endosperm, is expected to reduce the formation of incorrect glucose chains. This may decrease the need for trimming by HvISA1. As a result, the loss of HvBE2a has the potential to alleviate the phenotype of HvISA1.

Previous reports in barley RNAi experiments showed that silencing *HvBE2b* had less impact on amylopectin chain length distribution than *HvBE2a*. However, the double silencing of both genes exhibited an enhanced phenotype compared to the *HvBE2a* single silencing (Regina et al. 2010). This suggests a potential functional overlap or compensatory mechanism between *HvBE2a* and *HvBE2b*. In the *hvbe2a* mutant, we detected HvBE2b activity through Native-PAGE activity staining. Thus, HvBE2b is likely to function in the *hvbe2a* endosperm. It appears that HvBE2b has a minor role in creating inappropriate glucose chains in the endosperm, as there is a significant suppression of phytoglycogen production in *hvisa1-3 hvbe2a-1* and *hvisa1-3 hvbe2a-2* compared to the *hvisa1-3* single mutant (Fig. 5b). It is also possible that additional DBEs, such as pullulanase, play a crucial role in trimming the inappropriate glucose chains generated by HvBE2b, alongside ISA1. Notably, in rice, pullulanase has been proposed to be involved in debranching inappropriate glucose chains in *isa1*　mutant (Fujita et al. 2009).

The mutation in *BE* genes has been shown to alleviate the *isa1* phenotype in rice (Lee et al. 2017; Nagamatsu et al. 2022). Specifically, the *beIIb* rice mutation reduces the reduction in grain weight of *isa1* mutants and changes the glucan chain length distribution of α-glucan from the *isa1*-type to the *beIIb*-type (Nagamatsu et al. 2022). This result indicates that *beIIb* is epistatic to *isa1* in rice. Additionally, the *beIIa* mutation also can mitigate the phenotype caused by *isa1*, even though it shows minimal phenotype in rice (Satoh et al. 2003; Lee et al. 2017). These results indicate that the antagonistic relationship between *isa1* and *be* mutations is common in rice and barley.

The *hvisa1* mutant forms compound SGs, while *hvisa1-3 hvbe2a* double mutants develop bimodal simple SGs (Fig. 6a–i). This result suggests that in barley, mutations in the *HvISA1* and *HvBE2a* genes enable the conversion between compound and bimodal types of SGs. Soluble phytoglycogen molecules or their degradation products, such as maltooligosaccharides, are proposed to act as substrates for nucleating new granule initiation (Burton et al. 2002). The higher level of phytoglycogen in *hvisa1* mutants may increase the nucleation events and lead to more starch particles within an amyloplast, resulting in the formation of compound SGs (Burton et al. 2002). This idea is supported by the development of simple SGs instead of compound SGs in *hvisa1-3 hvbe2a* double mutants where the level of phytoglycogen was reduced (Figs. 5b, 6a–i). The fact that HvISA1 is not necessary for developing bimodal SGs under conditions with reduced HvBE2a activity suggests that HvISA1 does not play an exclusive function in developing bimodal SGs.

These mutations are unlikely to be involved in the natural variation in SG morphology across different plant species. For example, wild-type rice develops compound SGs, but the rice *beIIb* mutant increases the number of small spherical starch particles without forming the typical bimodal SGs observed in barley and wheat. The differences in glucan chain length distribution of amylopectin among species are not as pronounced as those caused by mutations in major starch biosynthetic enzymes (Jane et al. 1999). This suggests that the variation in SG morphology is influenced by factors other than mutations in starch biosynthetic enzymes.

### The minor impact of HvBE2a on starch granule formation in pollen

In cereal pollen, vegetative cells accumulate large amounts of starch (Lee et al. 2022). This starch acts as a nutritional source during pollen germination. Some starch-related genes expressed in pollen often function similarly in the endosperm. For instance, *waxy* mutants of rice, maize and sorghum, possessing mutations in the amylose-synthesizing enzyme, show an absence of amylose in both the endosperm and pollen (Terada et al. 2002; Pedersen et al. 2004; Talukder et al. 2022). The shape of SGs in pollen is commonly consistent among plant species, typically appearing as smaller, rod-shaped, and simple-type (Matsushima and Hisano 2019). The consistency of SG shapes in pollen contrasts with the wide range of SG morphologies found in the endosperm. Nevertheless, mutations affecting SG morphologies in the endosperm often lead to alterations in SG shape in pollen (Matsushima et al. 2014, 2016, 2023).

In the pollen of Haruna Nijo and the mutants, including *hvisa1-3*, *hvbe2a-1*, and *hvbe2a-2*, the ratio of compound SGs were less than 9%. (Fig. 8b). In contrast, the *hvflo6* pollen exhibited a significantly higher proportion of compound SGs, approximately 66.5% (Fig. 8b). Furthermore, the *hvflo6* phenotype was notably enhanced by the *hvisa1* mutation, resulting in over 90% compound SGs in the pollen (Fig. 8b). This suggests that the *HvISA1* gene plays a role in pollen, at least in the *hvflo6-2* background. The level of pollen compound SGs in *hvflo6-2 hvbe2a-1* and *hvflo6-2 hvbe2a-2* were found to be comparable to *hvflo6-2*, indicating no significant reduction. Similarly, there were no significant difference observed among *hvflo6-2 hvisa1-3 hvbe2a* triple mutants and *hvflo6-2 hvisa1-3* double mutants (Fig. 8b). These findings indicate that mutations in *hvbe2a* did not alleviate the *hvflo6-2 hvisa1-3* phenotype in pollen, which contrasts with the traits identified in the endosperm.

The level of HvBE2a accumulation in pollen was lower than in the endosperm (Fig. 8c). This suggests that HvBE2a has a less significant role in pollen compared to the endosperm. Notably, the accumulation of HvBE2b was increased in *hvflo6-2 hvbe2a-1*, *hvflo6-2 hvbe2a-2*, *hvflo6-2 hvisa1-3 hvbe2a-1*, and *hvflo6-2 hvisa1-3 hvbe2a-2* (Fig. 8d). All of these lines developed compound SGs in pollen despite having the *hvbe2a* mutations (i.e., the suppressive effect of the *hvbe2a* mutations is not observed). The HvBE2a depletion leads to the increase of HvBE2b, and the induced HvBE2b may compensate for the HvBE2a function (Fig. 8d). In the endosperm, due to its high dependency on HvBE2a, HvBE2b may not be able to fully compensate for the depletion of HvBE2a.

Another possible explanation for the different suppressive effects of HvBE2a depletion between pollen and endosperm is that the pathways leading to the increase of compound SGs differ between pollen and endosperm. In the endosperm, the depletion of HvISA1 leads to an increase in compound SGs (Fig. 6a, b). In contrast, in pollen, the depletion of HvISA1 does not increase compound SGs (Fig. 8a). This suggests that pollen does not require HvISA1 to maintain normal SG morphology. On the other hand, the absence of HvFLO6 leads to an increase in compound SGs in both pollen and endosperm. This indicates that there are independent pathways involving HvISA1 and HvFLO6 to maintain normal SG morphology in barley. Given that the compound SG increase in *hvflo6-2* pollen but not in *hvisa1-3*, it is likely that in wild-type pollen, the HvFLO6 pathway primarily ensures the formation of normal simple SGs in pollen. The suppressive effect of HvBE2a depletion on starch accumulation is not significant in *hvflo6-2* (Fig. 5a). This may explain why the suppressive effect of HvBE2a depletion is not observed in pollen, where the HvFLO6 pathway is dominant. However, even considering this possibility, it is difficult to explain why the suppressive effect of HvBE2a depletion is not observed in the *hvflo6 hvisa1* double mutant background. In the future, it will be necessary to create additional mutants of HvBE2b and HvBE1 in barley to investigate the contributions of these BEs in pollen.

## Conclusion

Mutations in starch-related genes play a crucial role in cereal breeding as they can affect taste, digestibility, and processing qualities. Cereals are versatile crops used for food, feed, malting, and industrial purposes, and their grain starch properties determine their suitability for these different applications. This study demonstrated that genetic interactions among specific mutations can control the amount and characteristics of starch and the SG morphology. Combining mutations to uncover genetic interactions that enhance or suppress traits are crucial for controlling starch properties. Multiple mutants could be used as breeding materials in future breeding efforts.

## Supplementary Information

Below is the link to the electronic supplementary material.

Supplementary file 1 (PDF 1147 KB)

## Data Availability

The sequence of HvBE2a, HvBE2b and HvBE1 are available in the Ensemble Plants database (http://plants.ensembl.org/index.html) as HORVU.MOREX.r3.2HG0165780.1, HORVU.MOREX.r3.2HG0170370.1 and HORVU.MOREX.r3.7HG0751660.1, respectively. The raw NGS sequence data are available in the DNA Data Bank of Japan Sequence Read Archive (DDBJ DRA) database under specific accession numbers PRJDB17596 for *hvbe2a-2* genome and PRJDB4103 for Haruna Nijo genome.

## Abbreviations

DAA: Days after awn emergence
DP: Degree of polymerization
HvBE2: Hordeum vulgare BRANCHING ENZYME2
HvISA1: Hordeum vulgare ISOAMYLASE1
HvFLO6: Hordeum vulgare FLOURY ENDOSPERM 6
SG: Starch granule
