# Supplementary material for: Mutations in starch BRANCHING ENZYME 2a suppress the traits caused by the loss of ISOAMYLASE1 in barley
Source: Theor Appl Genet. 2024 Aug 31;137(9):212. doi: 10.1007/s00122-024-04725-7 (PMC11365852; doi:10.1007/s00122-024-04725-7)
Supplement: Supplementary file 1 — (PDF 1147 KB) [file 122_2024_4725_MOESM1_ESM.pdf]

Supplementary Information

Supplementary Fig. 1 Measurement of grain sizes of *hvbe2a-1* and *hvbe2a-2*.
a-c Length, width and thickness of mature grains, respectively (n = 30, 30, 70, respectively). Statistical comparisons were performed using Welch's *t*-test (**, $p < 0.01$; *, $p < 0.05$;and ns, not significant at $p = 0.05$).

Supplementary Fig. 2 Plant appearance of the *hvbe2a-1* and *hvbe2a-2* mutants.
a-d Photographs of 28-day-old plants of Haruna Nijo (a), *hvbe2a-1* (b), *hvbe2a-2* (c). Bars = 5 cm. **d-e** Number of tillers and shoot fresh weight of 28-day-old plants (n = 3-5), respectively. Data are given as means ± SD. Statistical comparisons were performed using Welch's *t*-test (ns, not significant at $p = 0.05$). **f-h** Maturing panicles of Haruna Nijo, *hvbe2a-1*, *hvbe2a-2* at 20 days after awn emergence. Bar = 1 cm. **i-k** Mature panicles of Haruna Nijo, *hvbe2a-1*, *hvbe2a-2*. Bars = 1 cm.

Supplementary Fig. 3 PCR detection of the base change in *hvbe2a-1* mutant.
a To detect the *hvbe2a-1* mutation, PCR products were amplified using derived cleaved-amplified polymorphic sequence primers and digested with *Nco*I. In the case of the wild-type allele (Haruna Nijo), the PCR product (109 bp) was digested into fragments of 83 bp and 26 bp (the smaller band is not visible). The PCR product was not digested in the case of the *hvbe2a-1* allele. Heterozygous plants (*hvbe2a-1*$^{+/-}$) yielded both the full-length band and the digestion products. **b** The base changes in *hvbe2a-1* are co-segregated with *hvbe2a-1* elongated SG phenotype. Lanes indicated by numbers were the F2 seeds showing the existence of elongated SG in their endosperms. Closed and open arrowheads indicated the band positions specific to wild type and *hvbe2a-1* alleles, respectively.

Supplementary Fig. 4 Alignment of starch branching enzymes in barley.
Amino acid sequences of HvBE2a, HvBE2b and HvBE1 are aligned using Clustal W program. Perfectly conserved residues are shown in black. The synthetic peptides used for raising each antiserum are boxed.

Supplementary Fig. 5 Starch granules in *hvisa1-3 hvbe2a* mutant pollens.
a Iodine-stained SGs released from squashed pollen grains of *hvisa1-3*, *hvisa1-3 hvbe2a-1*, *hvisa1-3 hvbe2a-2*. Bars = 5 μm. **b** The percentage of starch granules in compound form, as determined from microscopic images. Data are given as means ± SD. The percentage of starch granules in *hvisa1-3* pollen was the same as that presented in Figure 8b. Statistical comparisons were performed using Welch's t-test (ns, not significant at $p = 0.05$).

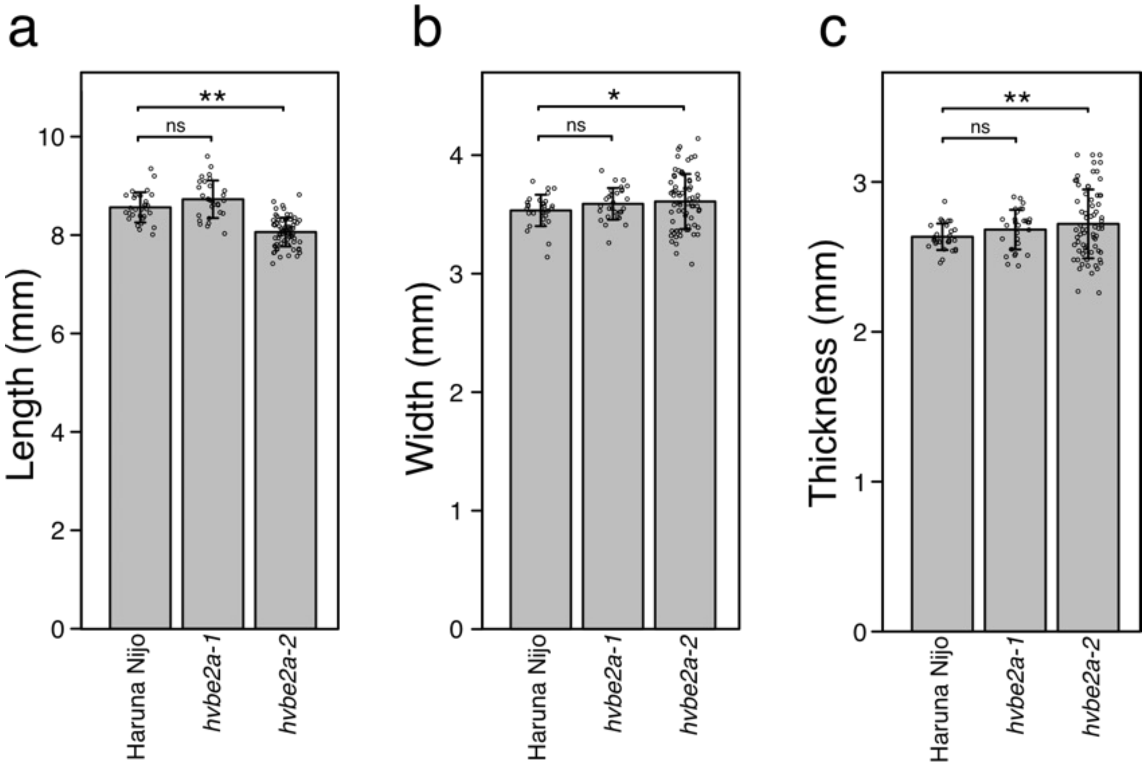

Supplementary Fig. 1 Measurement of grain sizes of *hvbe2a-1* and *hvbe2a-2*.

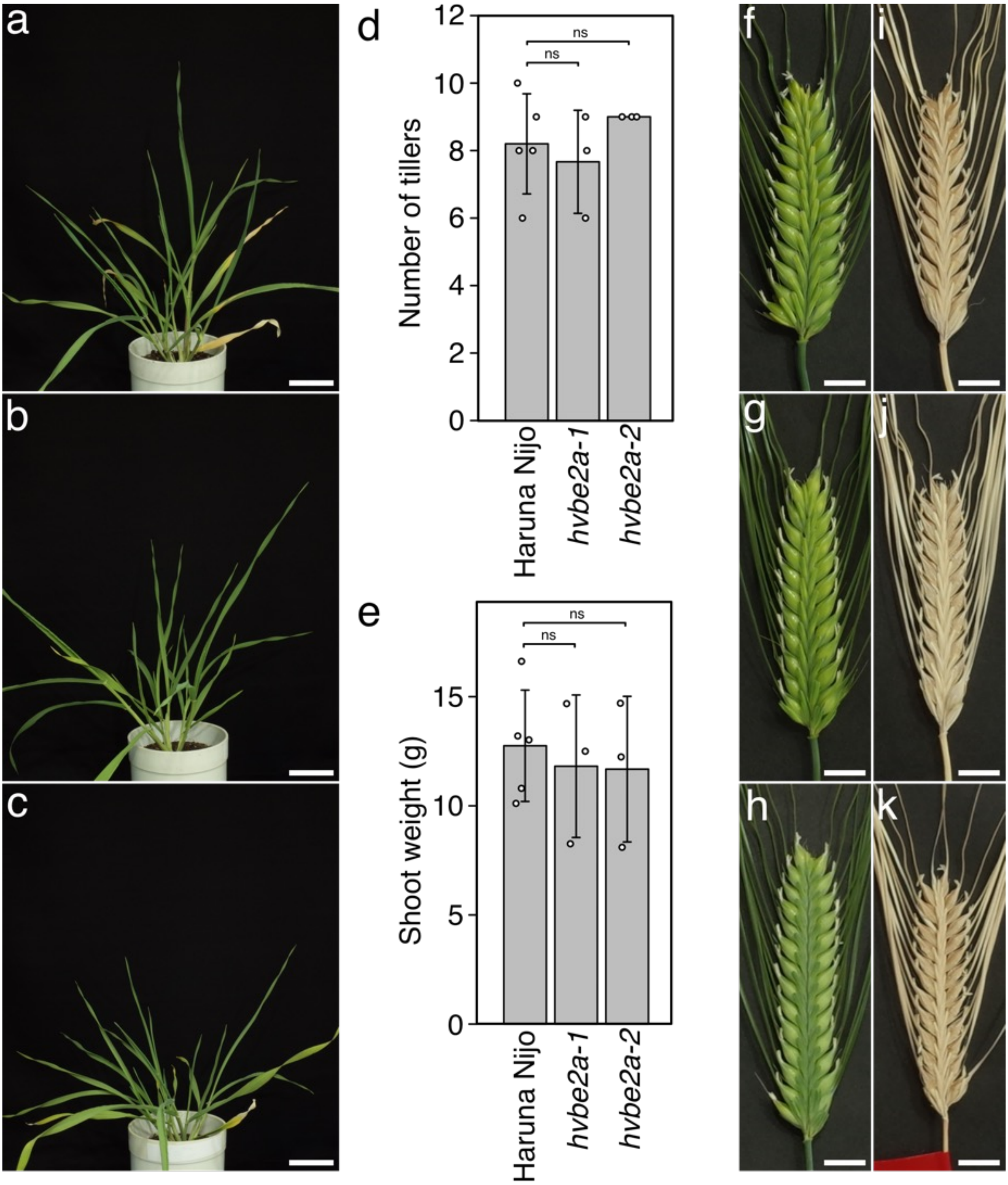

Supplementary Fig. 2 Plant appearance of the *hvbe2a-1* and *hvbe2a-2* mutants.

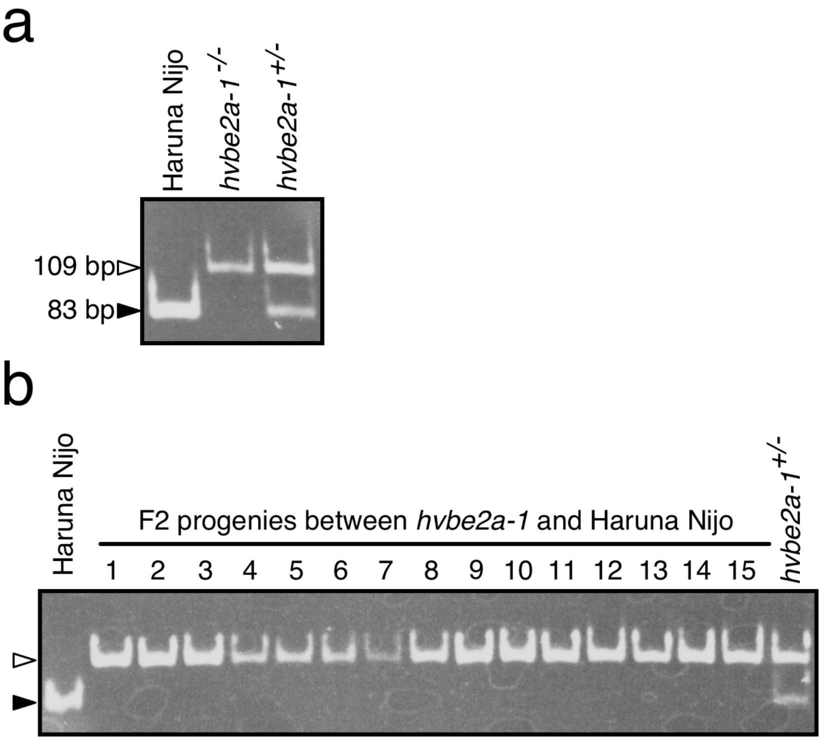

Supplementary Fig. 3 PCR detection of the base change in *hvbe2a-1* mutant.

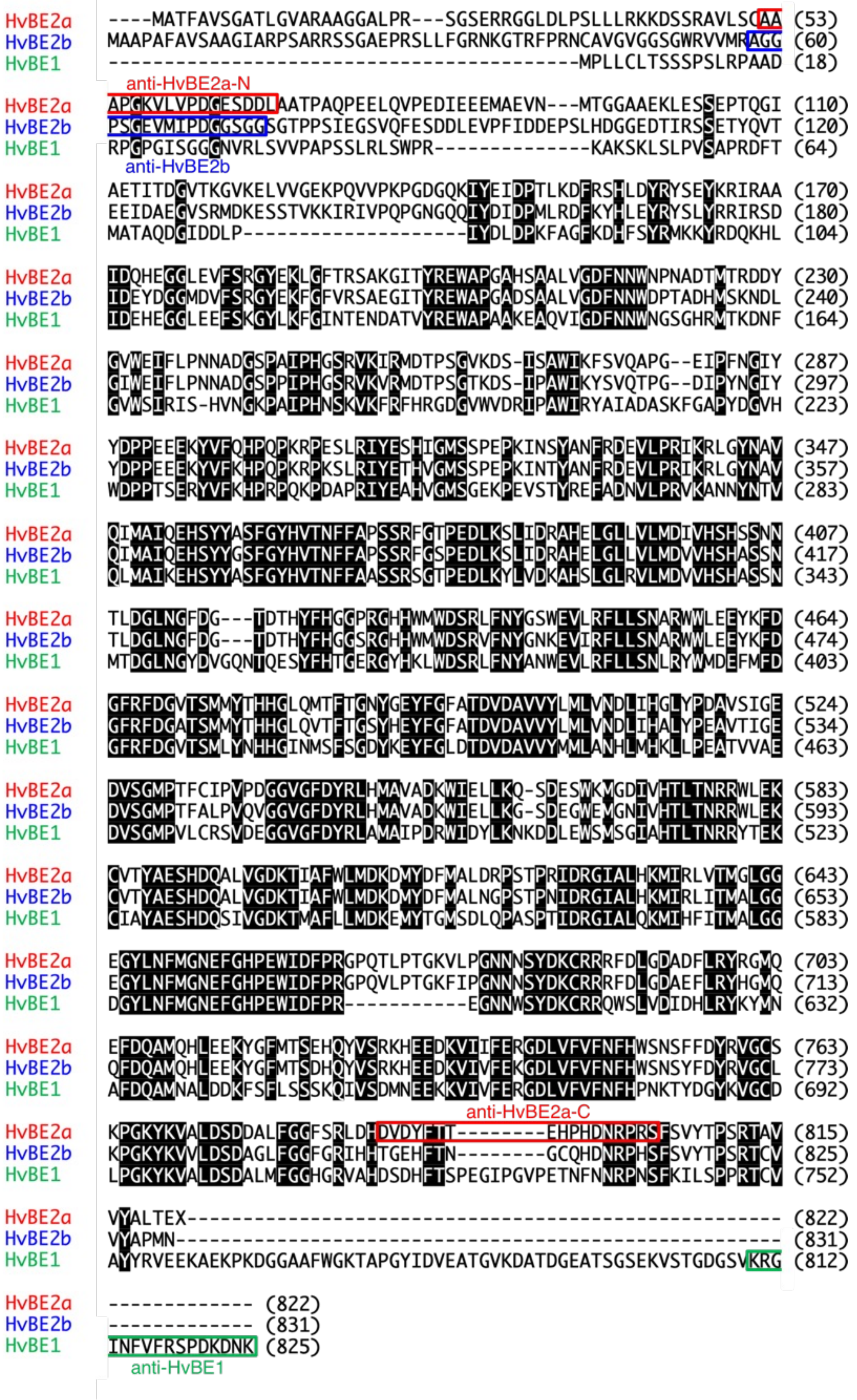

Supplementary Fig. 4 Alignment of starch branching enzymes in barley.

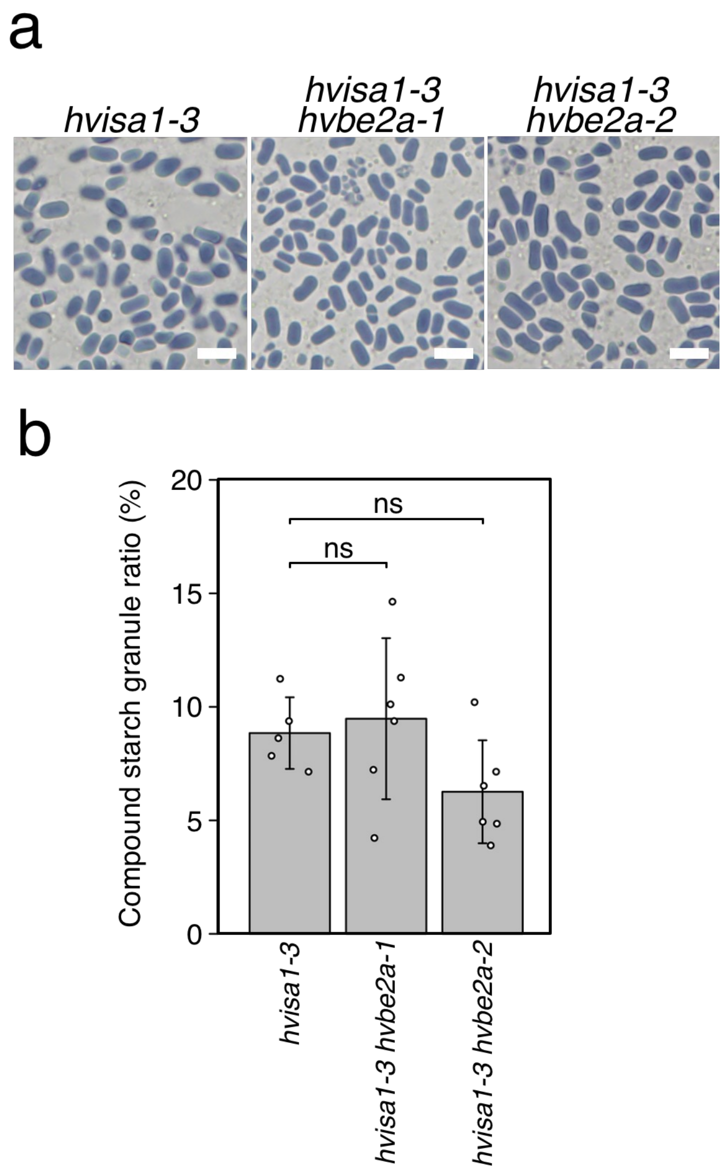

Supplementary Fig. 5 Starch granules in *hvisa1-3 hvbe2a* mutant pollens.
